# Manipulating chiral photon generation from plasmonic nanocavity-emitter hybrid systems: from weak to strong coupling

**DOI:** 10.1515/nanoph-2023-0738

**Published:** 2024-01-16

**Authors:** Jian Yang, Huatian Hu, Qingfeng Zhang, Shuai Zu, Wen Chen, Hongxing Xu

**Affiliations:** State Key Laboratory of Precision Spectroscopy, School of Physics and Electronic Science, East China Normal University, Shanghai 200241, China; Hubei Key Laboratory of Optical Information and Pattern Recognition, Wuhan Institute of Technology, Wuhan 430205, China; Center for Biomolecular Nanotechnologies, Istituto Italiano di Tecnologia, Via Barsanti 14, 73010 Arnesano (LE), Italy; College of Chemistry and Molecular Sciences, Wuhan University, Wuhan 430072, China; Advanced Photonics Center, School of Electronic Science and Engineering, Southeast University, Nanjing 210096, China; The Institute of Advanced Studies, School of Physics and Technology, Center for Nanoscience and Nanotechnology, and Key Laboratory of Artificial Micro- and Nano-structures of Ministry of Education, Wuhan University, Wuhan 430072, China

**Keywords:** chiral plasmons, chiral photon generation, high degree of circular polarization, strong coupling, chiral coupled oscillator model, chiral Jaynes–Cummings model

## Abstract

By confining light into a deep subwavelength scale to match the characteristic dimension of quantum emitters, plasmonic nanocavities can effectively imprint the light emission with unique properties in terms of intensity, directionality, as well as polarization. In this vein, achiral quantum emitters can generate chiral photons through coupling with plasmonic nanocavities with either intrinsic or extrinsic chirality. As an important metric for the chiral-photon purity, the degree of circular polarization (DCP) is usually tuned by various scattered factors such as the nanocavity design, the emitter type, and the coupling strategy. The physical mechanisms of the chiral photon generation, especially when plasmons and emitters step into the strong coupling regime, are less explored. In this paper, we extended the coupled-oscillator and Jaynes–Cummings models to their chiral fashion to account for the above factors within a single theoretical framework and investigated the chiroptical properties of a plasmonic nanocavity-emitter hybrid system from weak to strong coupling. It was demonstrated that both the circular differential scattering and prominent scattering DCP rely on the intrinsic chirality generated by breaking the mirror symmetry with the emitter, and is thereby tunable by the coupling strength. However, the luminescence DCP (as high as 87 %) is closely related to the extrinsic chirality of the bare nanocavity and independent of the coupling strength. The results thus reveal two different physical mechanisms of generating chiral photons in scattering and luminescence. Our findings provide a theoretical guideline for designing chiral photon devices and contribute to the understanding of chiral plasmon-emitter interaction.

## 1 Introduction

Bringing about new opportunities in increasing the optical information transmission and storage capacities [1], [2], chirality as an important degree of freedom of light has attracted substantial research interest in the past decades. Among the wide spectrum of photonic devices, plasmonic nanocavities with prominent Purcell effects [3], [4] and engineerable local chiral fields [5] can provide unique solutions for ultrafast chiral photon generation [6]. Specifically, plasmonic nanocavities can combine the large enhancement of photonic local density of states (LDOS) with either intrinsic (geometrical) chirality [7], [8], [9], [10], [11] or extrinsic (hidden) chirality [12], [13], [14] to create strong localized “chiral” hotspots where large differential LDOS between left- and right-handed-circularly-polarized (LCP/RCP) emission could be realized [13], [15]. These will allow chiral photon generation from the coupled plasmon-emitter systems in both scattering and luminescence processes. The chiral scattering (i.e. linear-to-circular polarization conversion) with a high degree of circular polarization (DCP) is critically important for the realization of nanoscale polarization converters [16], [17], [18]. On the other hand, chiral luminescence with a high DCP, a key ingredient for chiral photon sources, has been demonstrated in various kinds of systems including molecules [19], quantum dots [20], [21], nitrogen-vacancy centers [22], transition metal dichalcogenides (TMDC) [23], [24], and electron beams [13], [25], [26].

Understanding the physical mechanism of the interplay between chiral photon generation and plasmon-emitter coupling is of crucial importance for both fundamental knowledge and practical applications. For linear polarizations, it is well-known that plasmon-emitter interaction can lead to a plethora of intriguing physical phenomena ranging from Fano interference to Rabi splitting [27], [28], [29], and from enhanced luminescence to quantum coherent states [30], [31], [32], [33], [34]. Conventional studies on the interaction between “chiral” plasmonic nanocavities and quantum emitters often focus on the weak coupling (Purcell) regime [15]. More recently, chiral plasmon-emitter interaction in the strong coupling regime captured considerable attention, demonstrating Rabi splitting in the circular differential scattering (CDS) spectra [35] as well as interesting chiral plexcitonic states [36], [37], [38], [39], [40], [41], [42]. However, very few theoretical models have been developed to account for the chiral plasmon-emitter interaction [40], especially regarding the whole landscape of chiral photon generation in different coupling regimes from weak to strong.

**Figure 1:**
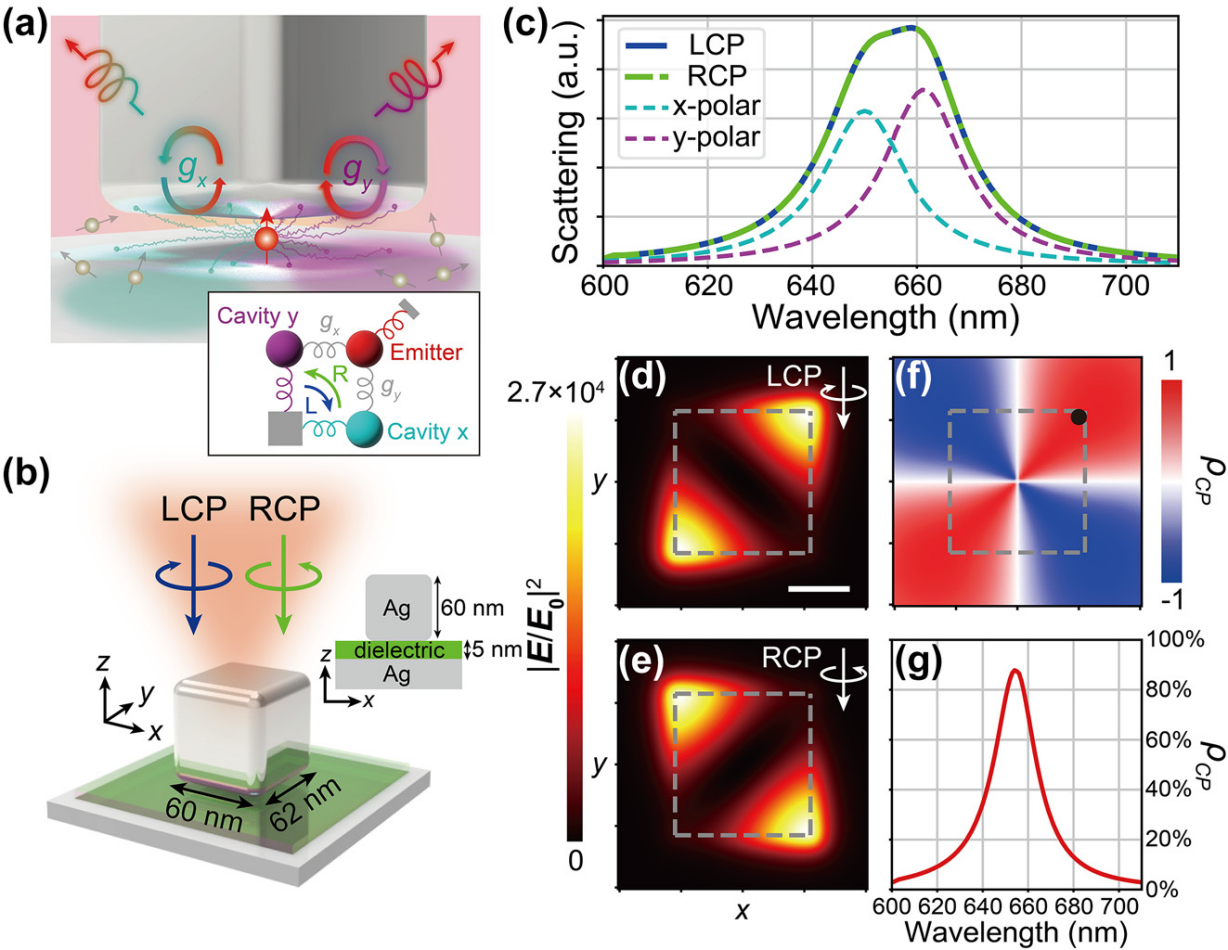
Extrinsic chirality of the bare NBoM cavity without quantum emitters. (a) Chiral photon generation from the NBoM-emitter hybrid system. The inset shows the schematic of the theoretical model. (b) 3D schematic of the NBoM cavity. The inset shows the 2D cross section in the *xz* plane. (c) Far-field scattering spectra under different incident light polarizations. (d)–(f) Field distribution ${\left|E/E_{0}\right|}^{2}$ under (d) LCP and (e) RCP excitations and (f) *ρ*_*CP*_ at the central plane of the dielectric spacer at the wavelength of 655 nm (between the resonant wavelengths of *x* and *y* plasmon modes). The scale bar in (d) is 20 nm. (g) *ρ*_*CP*_ at the black spot in (f) where the structure has optimal ${\left|E/E_{0}\right|}^{2}$ and *ρ*_*CP*_ simultaneously.

In this paper, we revealed the role played by the plasmon-emitter coupling in the CDS and chiral photon generation (i.e., chiral scattering and chiral luminescence) from a hybrid system composed of a plasmonic nanocavity and an achiral quantum emitter (Figure 1(a)) by combined theoretical formalisms and numerical methods. Specifically, we extended the coupled oscillator model (COM) and Jaynes–Cummings model (JCM) to their chiral fashion for a full description of the CDS, chiral scattering, and chiral luminescence in different coupling regimes from weak to strong. As a proof-of-concept testbed, the chiroptical properties of a hybrid structure consisting of a nanobar-on-mirror (NBoM) cavity with giant extrinsic chirality and a quantum emitter were studied. Distinguished features in scattering and luminescence were revealed: (i) Both the CDS and chiral scattering are induced by the intrinsic chirality generated by breaking the mirror symmetry of the nanocavity with the emitter. The coupling strength between the two components could reflect the degree of symmetry breaking and thereby engineer the chiroptical responses. (ii) The chiral luminescence DCP (as high as 87 % shown in results) is solely decided by the nanocavity’s local extrinsic chirality and independent of the coupling strength. While prominent Rabi splitting is present in the LCP/RCP luminescence spectra, there is no splitting in the DCP spectrum. Overall, our work proposes a set of concise formalisms that work as useful tools for manipulating and optimizing the chiroptical properties of chiral photon devices, and brings new insights into the different physical mechanisms of chiral photon generation in scattering and luminescence.

## 2 Results and discussion

### 2.1 Extrinsic (hidden) chirality in the bare NBoM antenna

Since the chiroptical effects originate from the coupling between the achiral emitter and plasmonic nanocavity with only extrinsic chirality, we first investigate the crucial extrinsic chiroptical properties of the bare plasmonic component. Here, we chose a versatile NBoM cavity [15] from the nanoparticle-on-mirror family [43], [44], [45], [46] as a proof-of-concept platform because of its remarkable extrinsic chirality, extremely large photonic LDOS, as well as outstanding directional emission pattern as a nanopatch antenna [3], [47], [48]. The schematic of the NBoM cavity is shown in Figure 1(b): a silver nanobar is placed on a dielectric spacer on top of a silver mirror.

Although the NBoM cavity is intrinsically achiral due to its mirror symmetry, it is endowed with an extrinsic chirality by asymmetric excitation. The “extrinsic” chirality, or sometimes termed “hidden” chirality in previous literatures [12], [13], [25], [26], [49], [50], [51], [52], can be generated extrinsically by asymmetric excitation conditions such as oblique incidence [12], [49], circularly-polarized incidence [50], [51], [52], or local sources at designed positions [25], [26]. This combination of achiral nanostructures and asymmetric excitation breaks the mirror symmetry and harbors chiroptical properties. Extrinsic chirality often involves the interference between two degeneracy-lifted plasmon modes [14], [52], inducing circular differential local near-field distribution for interacting with quantum emitters. Following this methodology, our nanobar is deliberately designed with dimensions of 60 × 62 × 60 nm^3^ to introduce degeneracy lifting along the *x* and *y* axes. The extrinsic chirality of our system becomes evident under circularly-polarized incidence or excitation by local sources at specific positions. As shown in the Supplementary Material S4, a slight degeneracy lifting can produce the desired extrinsic chirality, with the optimal circular differential responses achieved with a 2 nm difference in lengths. Nevertheless, the structure has a relatively large tolerance on the deviation of the nanobar size (Supplementary Material S5).

Figure 1(c) shows the far-field scattering spectra of the bare NBoM cavity under different incident polarization states. For LCP and RCP excitations, the scattering spectra are identical and correspond to the superposition of two dipolar modes in the *x* and *y* directions with a small offset due to the degeneracy lifting. This confirms that the NBoM is indeed intrinsically an achiral nanostructure due to the evident mirror symmetry in geometry. While the overall integral scattering is identical for LCP and RCP excitations, the far-field scattering pattern shows noticeable angular dependence (Supplementary Material S6). Moreover, at a wavelength between the resonant wavelengths of *x* and *y* dipolar modes (i.e., at 655 nm here), the electric fields at the central *x*–*y* cross-section of the nanogap are distributed at the cube corners along two different diagonals (Figure 1(d) and (e)). This phenomenon can be explained by a harmonic oscillator model where the phase difference between the *x* and *y* dipolar modes induces either constructive or destructive interference in the electric near field (Supplementary Material S7).

To quantify the local chirality displayed under LCP and RCP excitations, we define the circular differential local electric field as $\rho_{CP}=\left({\left|E_{L}\right|}^{2}-{\left|E_{R}\right|}^{2}\right)/\left({\left|E_{L}\right|}^{2}+{\left|E_{R}\right|}^{2}\right)$ . Where ***E***_**L/R**_ are the electric fields under LCP and RCP excitations, respectively. As shown in Figure 1(f), *ρ*_*CP*_ could approach unity with either left- or right-handed chirality. At the black spot denoted in Figure 1(f), the nanocavity shows simultaneously the largest electric field enhancement (Figure 1(d)) and the greatest *ρ*_*CP*_ of 87 % (Figure 1(g)). According to the principle of reciprocity [53], by introducing a quantum emitter at such point as a local source, we can take advantage of the large enhancement and extrinsic chirality to achieve ultrabright and high-purity circularly-polarized emission. Again, we point out that both the field enhancement and *ρ*_*CP*_ are tolerant to the deviation in the position of the quantum emitter (Supplementary Material S5). It is worth mentioning that the extrinsic chirality only characterizes the circular differential electric field distribution under LCP and RCP excitations. It is not directly connected with the optical chirality which measures the local field chirality (Supplementary Material S8).

### 2.2 Chiroptical responses in the scattering of the hybrid system from weak to strong coupling

**Figure 2:**
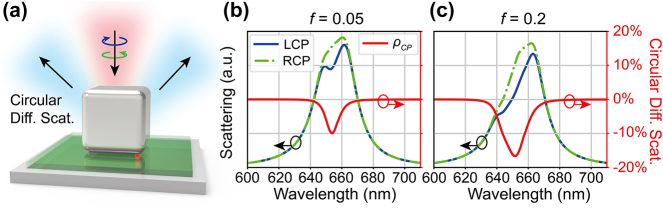
Modulation of CDS by the plasmon-emitter coupling predicted by numerical simulations. (a) Schematic of the CDS of the NBoM-emitter hybrid system. (b) and (c) simulated scattering spectra under LCP/RCP excitations (blue/green lines) and CDS spectra (red lines) for the NBoM-emitter hybrid system for (b) emitter oscillator strength *f* = 0.05 and (c) *f* = 0.2.

Although the NBoM cavity possesses prominent extrinsic chirality, this extrinsic chirality does not generate any chiroptical response in the far-field scattering (Figure 1(c)). By introducing a quantum emitter into the nanocavity at the position shown in Figure 1(f), the mirror symmetry of the nanocavity can be broken (Supplementary Material S9), physically allowing the generation of chiral responses. Figure 2(a) shows the schematic of the NBoM-emitter hybrid system under LCP or RCP excitation. We first performed numerical simulations to study the CDS of the hybrid system for different coupling strengths. The quantum emitter was modelled as a small nanocylinder embedded in the dielectric spacer with a Lorentzian function permittivity $\varepsilon_{em}\left(\omega \right)=\varepsilon_{\infty }-f\omega_{em}^{2}/\left(\omega^{2}-\omega_{em}^{2}+i\Gamma_{em}\omega \right)$ , where *ɛ*_∞_ is the emitter background permittivity, *ω*_*em*_ and Γ_*em*_ are angular frequency (energy) and damping rate (FWHM) of the emitter transition mode. The oscillator strength *f* characterizes the plasmon-emitter coupling strength [54]. Experimental realization of similar kinds of hybrid nanostructures has been achieved by embedding TMDC flakes [25], [55], molecules [3], [56], or quantum dots [47], [57], [58] under plasmonic nanoparticles using advanced nanofabrication techniques. However, the chiroptical responses especially regarding the CDS and chiral scattering have rarely been explored.

Interestingly, the CDS manifests in this hybrid system and is tunable by the plasmon-emitter coupling. The CDS has been an important metric for the chiroptical properties of nanostructures [59]. It is defined as $\rho_{CP}=\left(Q_{\mathrm{scat,L}}-Q_{\mathrm{scat,R}}\right)/\left(Q_{\mathrm{scat,L}}+Q_{\mathrm{scat,R}}\right)$ where *Q*_*scat,L∕R*_ are the scattering cross sections for LCP/RCP excitations. As shown in Figure 2(b) and (c), with the increase of the oscillator strength *f* which indicates growing plasmon-emitter interaction strength, the far-field scattering spectra for both LCP and RCP excitations show more pronounced modes splitting and larger absolute CDS values. This result clearly demonstrates that the NBoM-emitter coupling induces far-field chirality in the hybrid structure which has a critical dependence on the coupling strength. The angular distribution of CDS is shown in the Supplementary Material S6. However, the far-field extinction spectra show little difference between LCP and RCP excitations, indicating an opposite-sign circular dichroism in absorption which compensates that in scattering (Supplementary Material S11).

To understand and further utilize this coupling mechanism, we proposed a chiral-COM which extends the classical COM [60] to account for chirality. Circular polarization can be decomposed into two orthogonal linear polarizations with ±π/2 phase lag. The chiroptical response of the coupled system arises from the phase difference between the *x* and *y* plasmon modes, and thereby can be tuned by their magnitudes and phases. The chiral-COM borrows the essence of the plasmonic Born–Kuhn model [61] which models the interference between two orthogonally-polarized plasmon modes using their relative phase difference. It is capable of modeling coupled systems that comprise a quantum emitter and two orthogonally-polarized plasmon eigenmodes. While the two plasmon modes may not exhibit direct coupling with each other, they can indirectly interfere through their respective coupling with the quantum emitter. The phenomenological chiral-COM model is intuitive and intrinsically contains all the information about the coupling components and their interaction. A schematic of the model is shown in the inset of Figure 1(a). In our case, the NBoM cavity supports two orthogonal plasmon eigenmodes along the *x* and *y* directions. These two directly uncoupled modes are both coupled to the emitter through the coupling strengths *g*_*x*_ and *g*_*y*_, respectively. The circularly polarized incident excitation can be modelled by two orthogonal driving forces *F*_*x*_ and *F*_*y*_ which have a phase lag Δ*φ* = ±π/2. For the chiral-COM, the equations of motion are as follows:

(1) $$\begin{array}{c} {\ddot{\mu }}_{x}+\Gamma_{\text{x}}{\dot{\mu }}_{x}+\omega_{x}^{2}\mu_{x}=F_{x}-2g_{x}{\dot{\mu }}_{em} \end{array}$$

(2) $$\begin{array}{c} {\ddot{\mu }}_{y}+\Gamma_{y}{\dot{\mu }}_{y}+\omega_{y}^{2}\mu_{y}=F_{y}-2g_{y}{\dot{\mu }}_{em} \end{array}$$

(3) $$\begin{array}{c} {\ddot{\mu }}_{em}+\Gamma_{\text{em}}{\dot{\mu }}_{em}+\omega_{em}^{2}\mu_{em}=2g_{x}{\dot{\mu }}_{x}+2g_{y}{\dot{\mu }}_{y} \end{array}$$

where *μ*_*x*,*y*,*em*_ are the electric dipole moments, *ω*_*x*,*y*,*em*_ are the mode angular frequencies (energies), and Γ_*x*,*y*,*em*_ are the dissipation rates (FWHM) of the plasmon *x*, *y*, and emitter modes, respectively. $F_{x}=F_{0}\exp\left(-i\omega t\right)$ and $F_{y}=F_{0}\exp\left(-i\omega t\pm \pi /2\right)$ are the external driving fields along the *x* and *y* directions. A factor of 2 before the coupling strengths *g*_*x*,*y*_ accounts for the different conventions used between the chiral-COM and the following chiral-JCM description. Thus, conventional scattered ways of tuning the chiroptical properties by nanocavity design, emitter type, and coupling strategy can be incorporated into a unified theoretical model: nanocavity designs are accounted for by mode energies *ω*_*x*,*y*_, mode damping rates Γ_*x*,*y*_ and vacuum electric field ***E***_***vac***_, emitter types are accounted for by emitter transition energy *ω*_*em*_, emitter damping Γ_*em*_, and transition dipole moment ***μ***_0_, and coupling strategies are represented by the coupling strength *g* = ***μ***_0_⋅ ***E***_***vac***_.

Analytical solutions of the dipole moments can be easily obtained by solving Equations (1)–(3):

(4) $$\begin{array}{c} \mu_{x}\left(\omega \right)=F_{0}\frac{L_{y}\left(\omega \right)L_{em}\left(\omega \right)-4\omega^{2}g_{y}^{2}\pm 4i\omega^{2}g_{x}g_{y}}{L_{x}\left(\omega \right)L_{y}\left(\omega \right)L_{em}\left(\omega \right)-4L_{x}\left(\omega \right)\omega^{2}g_{y}^{2}-4L_{y}\left(\omega \right)\omega^{2}g_{x}^{2}} \end{array}$$

(5) $$\begin{array}{l} \mu_{y}\left(\omega \right)=\pm iF_{0}\frac{L_{x}\left(\omega \right)L_{em}\left(\omega \right)-4\omega^{2}g_{x}^{2}\mp 4i\omega^{2}g_{x}g_{y}}{L_{x}\left(\omega \right)L_{y}\left(\omega \right)L_{em}\left(\omega \right)-4L_{x}\left(\omega \right)\omega^{2}g_{y}^{2}-4L_{y}\left(\omega \right)\omega^{2}g_{x}^{2}} \end{array}$$

where $L_{x,y,em}\left(\omega \right)=\omega_{x,y,em}^{2}-\omega^{2}-i\Gamma_{x,y,em}\omega$ are the Lorentzian denominators. The last terms in the numerators ±4*iω* ^2^ *g*_*x*_ *g*_*y*_ are the cross-coupling terms which correspond to the coupling from the other orthogonal mode through the emitter. These cross-coupling terms are sign-reversed for LCP/RCP excitations and proportional to *g*_*x*_ *g*_*y*_, clearly revealing the mechanism of generating the chiroptical response: the originally-uncoupled cavity *x* and *y* modes couple through the emitter and obtain either constructive or destructive interference effect for LCP or RCP excitations, with the interference strength determined by the coupling strength. We further deducted the analytical expression for the CDS (Supplementary Material S13), which serves as a design rule for optimizing the hybrid nanostructure for improved chiroptical responses:

(6) $$\begin{array}{c} \rho_{CP}=g_{x}g_{y}\left[\left(\omega_{x}^{2}-\omega_{y}^{2}\right)\Gamma_{em}+\left(\omega_{em}^{2}-\omega^{2}\right)\left(\Gamma_{x}-\Gamma_{y}\right)\right]/\tilde{A} \end{array}$$

where the denominator $\tilde{A}$ is a complex expression depending on the nanocavity, emitter, and coupling properties. Nevertheless, a straightforward conclusion can be reached from Equation (6): the CDS is nonzero only when the plasmonic nanocavity is coupled to the emitter (*g*_*x*_ *g*_*y*_ ≠ 0) and the degeneracy between the plasmon modes *x* and *y* is lifted (symmetry breaking, $\left(\omega_{x}^{2}-\omega_{y}^{2}\right)\left(\Gamma_{x}-\Gamma_{y}\right)\neq 0$ ).

Recently, it was proposed that the quantum Jaynes–Cummings model [62] was able to compare scattering, absorption, and luminescence on equal footing [63]. We also extended the JCM to chiral-JCM to model the CDS for the sake of both verification and universality. Moreover, the chiral-JCM will be useful for the investigation of chiral luminescence in the next section. The Hamiltonian under monochromatic circularly-polarized driving field within the rotating wave approximation can be written as [64]:

(7) $$\begin{array}{ll} H & =\Delta_{x}a_{x}^{†}a_{x}+\Delta_{y}a_{y}^{†}a_{y}+\Delta_{em}\sigma_{+}\sigma_{-}+g_{x}\left(a_{x}^{†}\sigma_{-}+a_{x}\sigma_{+}\right)\\ & \;+g_{y}\left(a_{y}^{†}\sigma_{-}+a_{y}\sigma_{+}\right)+\frac{1}{2}\left(\varepsilon_{\text{x}}a_{x}^{†}+\varepsilon_{\text{x}}^{*}a_{x}\right)\\ & \;+\frac{1}{2}\left(\varepsilon_{\text{y}}a_{y}^{†}+\varepsilon_{\text{y}}^{*}a_{y}\right) \end{array}$$

where Δ_*x*,*y*,*em*_ = *ω* − *ω*_*x*,*y*,*em*_ is the energy detuning between excitation and the three modes, *a*_*x*,*y*_ and $a_{x,y}^{†}$ are the annilation and creation operators of the *x* and *y* plasmon modes, *σ*_+,−_ are the emitter Pauli operators, *ɛ*_*x*,*y*_ are the external driving field operators and *ɛ*_*y*_ = ±*iɛ*_*x*_ for circularly-polarized excitation. Details can be found in the Supplementary Material S3.

**Figure 3:**
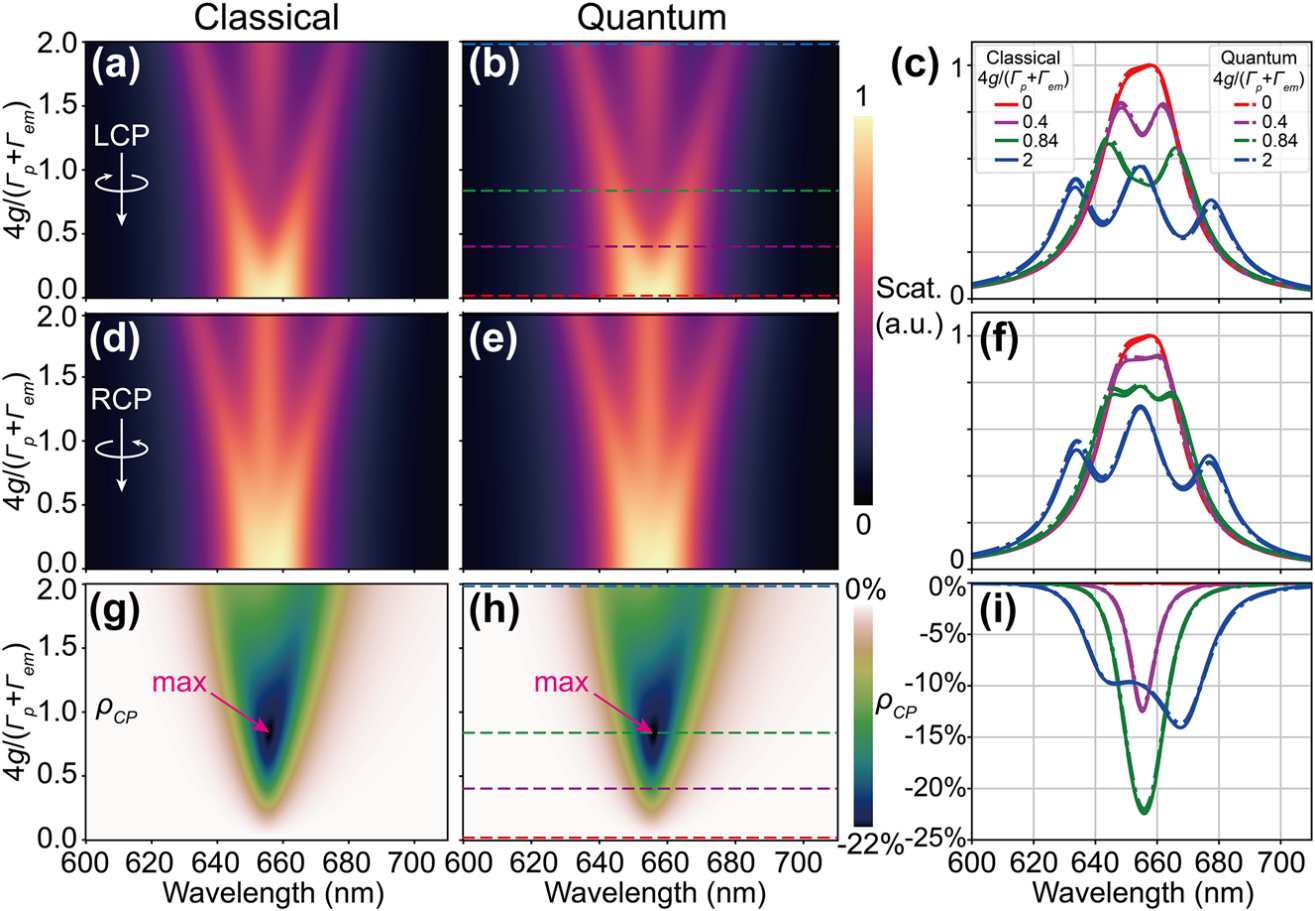
Modulation of CDS by the plasmon-emitter coupling predicted by classical and quantum models. (a)–(f) scattering mapping with respect to wavelength and coupling strength for LCP and RCP excitations based on chiral-COM and chiral-JCM. (c) and (f) show characteristic scattering spectra for four different coupling strengths. (g)–(i) CDS predicted by different models. The pink arrows in (g) and (h) indicate maximum CDS magnitudes.

As shown in Figure 3, both the chiral-COM (classical) and chiral-JCM (quantum) can model the CDS behavior of the hybrid system for different coupling strengths, showing nearly identical results that verify each other. For our NBoM cavity, the degeneracy lifting between the *x* and *y* plasmon modes is very small. At the emitter position, *g*_*x*_ and *g*_*y*_ only differ by 3 %. Thus, we introduced an average coupling strength as the geometric mean $g=\sqrt{g_{x}g_{y}}$ to eliminate the necessity of explicitly specifying *g*_*x*_ and *g*_*y*_ individually. For both LCP and RCP excitations, as the coupling strength increases, the modes splitting gets larger and the scattering spectra evolve from initial two overlapping peaks at weak coupling to the final three pronounced peaks at strong coupling (Figure 3(a)–(f)). These observations are in good agreement with the simulated spectral evolution with the increase of the emitter oscillator strength *f* (Figure 2). Major differences between the LCP and RCP excitations lie at the intermediate coupling regime where the LCP spectra are dim at the emitter wavelength (655 nm) while the RCP spectra are bright. This prominent CDS is due to the aforementioned destructive/constructive interference between the *x* and *y* plasmon modes through the emitter for LCP/RCP excitations. Figure 3(g)–(i) illustrate the evolution and modulation strategy of the CDS: *ρ*_*CP*_ increases with the coupling strength and reaches its optimum at intermediate coupling 4*g*/(Γ_*p*_ + Γ_*em*_) = 0.84, while further increase in coupling strength leads to splitting in the *ρ*_*CP*_ spectra and reduction of its magnitude. Although we show in Equation (6) that the numerator of *ρ*_*CP*_ is proportional to *g* ^2^, the denominator $\tilde{A}$ contains a *g* ^4^ term which dominates at strong coupling and reduces the magnitude of *ρ*_*CP*_. Physically, this comes from the fact that only a sub-component of the plasmon mode *x* or *y* that is independent of the coupling strength is interfering with the cross-coupling term from the other orthogonally-polarized plasmon mode through the emitter (Supplementary Material S13).

Interestingly, the theoretical extinction spectra also turn out to have no circular dichroism under circularly-polarized excitation, which is again consistent with the numerical simulations (Supplementary Material S15). Mathematically, the effects of the cross-coupling terms in the dipole moments *μ*_*x*,*y*_ (Equations (4) and (5)) on the overall extinction cancel out each other. For LCP excitation, constructive interference between the *x* and *y* cavity modes results in a significant amount of energy being channeled into the emitter and eventually dissipates non-radiatively. Therefore, the emitter dipole moment and emitter absorption under LCP excitation are larger than those under RCP excitation. On the other hand, RCP excitation leads to destructive interference at the emitter position, causing most of the energy to be scattered. This interesting observation indicates that solely measuring the scattering or extinction spectrum, as was typically done in experimental studies, might not give a complete picture of the chiroptical properties of the coupled systems.

**Figure 4:**
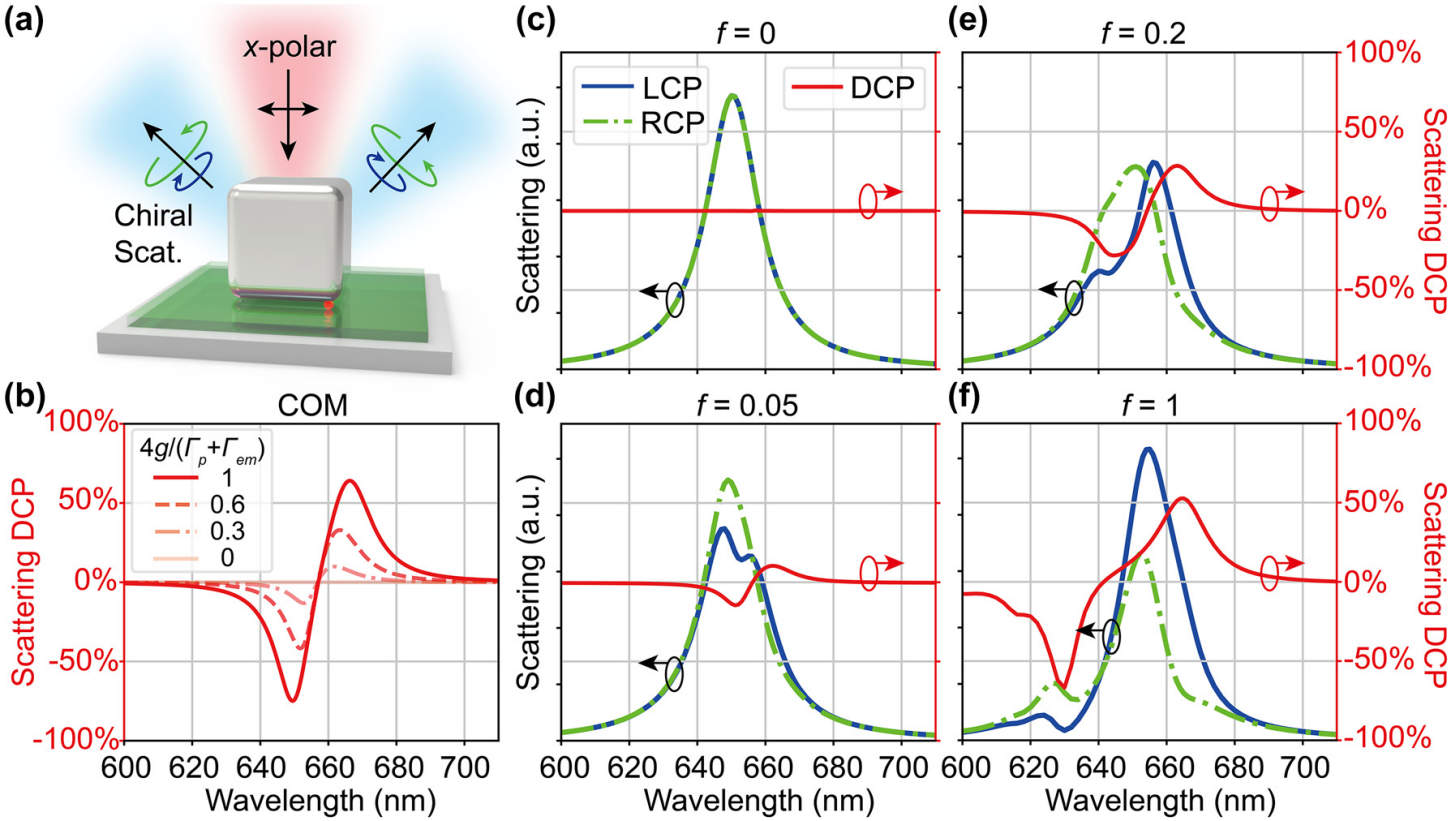
Modulation of chiral scattering DCP by the plasmon-emitter coupling. (a) Schematic of chiral scattering from the plasmon-emitter hybrid system under *x*-polarized excitation. (b) Dependence of scattering DCP spectra on the coupling strength calculated using the COM. (c)–(f) Numerical simulation of the chiral scattering from the hybrid system for emitter oscillator strength (c) *f* = 0, (d) *f* = 0.05, (e) *f* = 0.2, and (f) *f* = 1.

The above results clearly demonstrate that breaking the mirror symmetry of the NBoM cavity by coupling with a quantum emitter at the nanogap corner leads to the CDS behavior. Similarly, this symmetry breaking can also generate chiral scattering (scattered light with nonzero DCP) under linearly-polarized excitation (Figure 4(a)). Linear-to-circular polarization conversion has previously been demonstrated in bulky multi-layer stacked surfaces or large metasurfaces [16], [17], [18]. However, such conversion based on plasmonic nanocavity-emitter coupling was less explored, which can be important for the realization of nanoscale on-chip polarization converters.

Figure 4(b) shows the scattering DCP of our plasmon-emitter hybrid system under *x*-polarized excitation as a function of the coupling strength calculated using the COM. Here, the scattering DCP is defined as $DCP=\left(I_{L}-I_{R}\right)/\left(I_{L}+I_{R}\right)$ , where *I*_*L*/*R*_ are the intensities of the LCP and RCP components of the outgoing scattered wave. For zero coupling, the mirror symmetry is preserved and the scattering DCP remains zero. With the increase of the coupling strength, the scattering DCP shows a bisignate spectral lineshape with growing absolute values: either positive or negative DCP can be obtained at different wavelength regimes. For normalized coupling strength $4g/\left(\Gamma_{p}+\Gamma_{em}\right)=1$ , the DCP magnitudes reach over 60 %. Similar to the CDS, the chiral scattering originates from the indirect coupling between the two orthogonal plasmon modes through their respective coupling with the emitter. The incident *x*-polarized light first excites the *x* plasmon mode, which pumps energy into the emitter through *x* plasmon-emitter coupling *g*_*x*_. Then the emitter excites the *y* plasmon mode through the *y* plasmon-emitter coupling *g*_*y*_. The excited *y* plasmon mode can have a phase shift unequal to 0 or *π* relative to the *x* plasmon mode, generating chiral (elliptically-polarized) scattering with nonzero DCP (unequal LCP and RCP components). Since the amplitude and phase of the *y* plasmon mode are highly dependent on the coupling strength, the chiral scattering DCP is tunable by the plasmon-emitter coupling. Figure 4(c)–(f) show the corresponding numerical simulation results which are in good agreement with the COM. The angular distribution of chiral scattering DCP is shown in the Supplementary Material S17. Our results clearly demonstrate that chiral scattering can be manipulated by tuning the plasmon-emitter coupling, serving as an ideal platform for nanoscale linear-to-circular polarization conversion.

### 2.3 Chiral luminescence of the hybrid system from weak to strong coupling

The generation and modulation of chiral luminescence from quantum emitters through the manipulation of engineered electromagnetic vacuum field play key roles in the development of chiral photon sources [6], [20], [22]. Specifically, chiral emission can be enabled and manipulated by adjusting the differential radiative LDOS between LCP and RCP light. In the case of our NBoM cavity, the extrinsic chirality has the ability to modulate the inherently achiral emission from a quantum emitter positioned within the nanocavity. According to the principle of reciprocity, the emission from a local dipole at the nanobar corner should exhibit the same amount of chirality as the circular differential local ${\left|E/E_{0}\right|}^{2}$ (Figure 1(f) and (g)) [16]. As shown in the Supplementary Material S18, numerical simulations suggest that an achiral point dipole polarized along the *z*-direction generates luminescence with a high DCP. This local chirality can result in a non-trivial effect when coupled with quantum emitters, as these emitters typically possess inhomogeneous spatial distributions and unknown polarization directions within the nanocavity (Figure 1(a)). Recently, it has been demonstrated that luminescence can possess different handedness in different emission directions [65], [66]. Nevertheless, for our nanostructure, the chiral luminescence DCP is almost constant across a wide range of emission directions (Supplementary Material S19), which can be advantageous for the design of robust chiral photonic sources.

**Figure 5:**
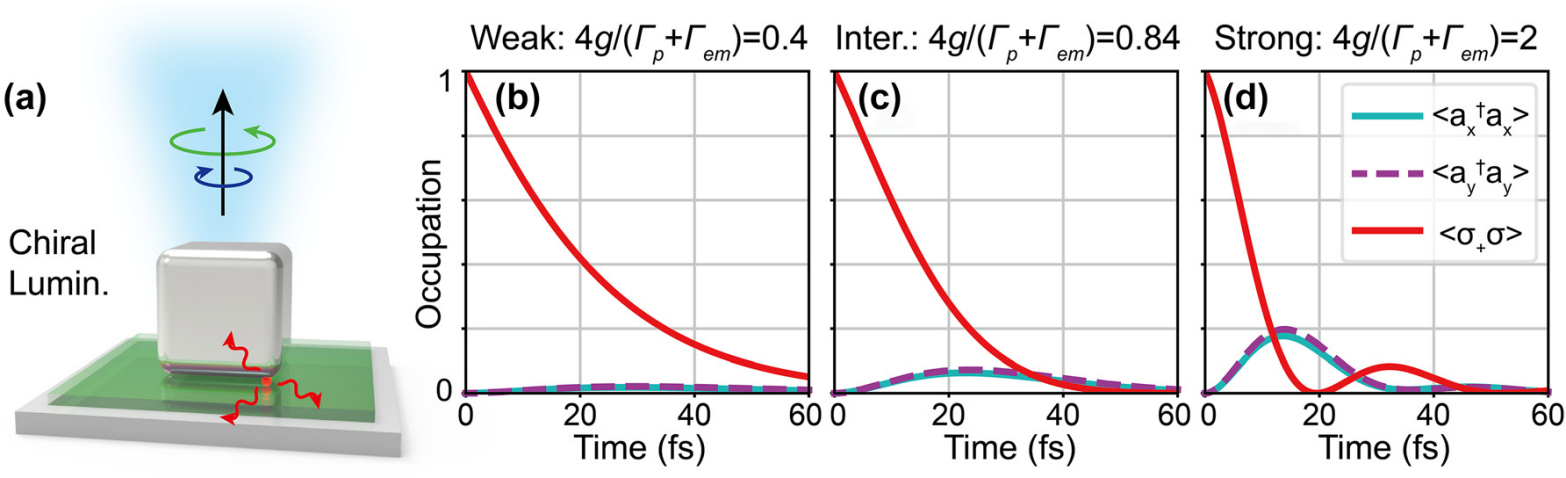
Emitter damping for different plasmon-emitter coupling strengths. (a) Schematic of the emitter decay and chiral luminescence. (b)–(d) Evolution of *x*, *y* plasmon and emitter occupation with an initial excited emitter for (b) weak, (c) intermediate, and (d) strong coupling calculated based on chiral-JCM.

To explore the plasmon-emitter interaction mechanism in chiral luminescence from weak to strong coupling, we study the chiral luminescence from the NBoM-emitter hybrid structure by the chiral-JCM (see Supplementary Material S20 for chiral-COM). The decay of an excited emitter inside the nanocavity was first investigated, as shown in Figure 5(a). The evolution of *x*, *y* plasmon and emitter occupations ( $a_{x}^{†}a_{x}$ , $a_{y}^{†}a_{y},\sigma_{+}\sigma_{-}$ ) for different coupling strengths is shown in Figure 5(b)–(d). For weak coupling, the energy stored in the emitter decays out without significant excitation of the plasmon modes (Figure 5(b)). However, for intermediate coupling, noticeable excitation of the cavity modes can be observed (Figure 5(c)). The Rabi oscillations, characteristic of strong coupling, are clearly visible in Figure 5(d) for a normalized coupling strength of $4g/\left(\Gamma_{p}+\Gamma_{em}\right)=2$ .

**Figure 6:**
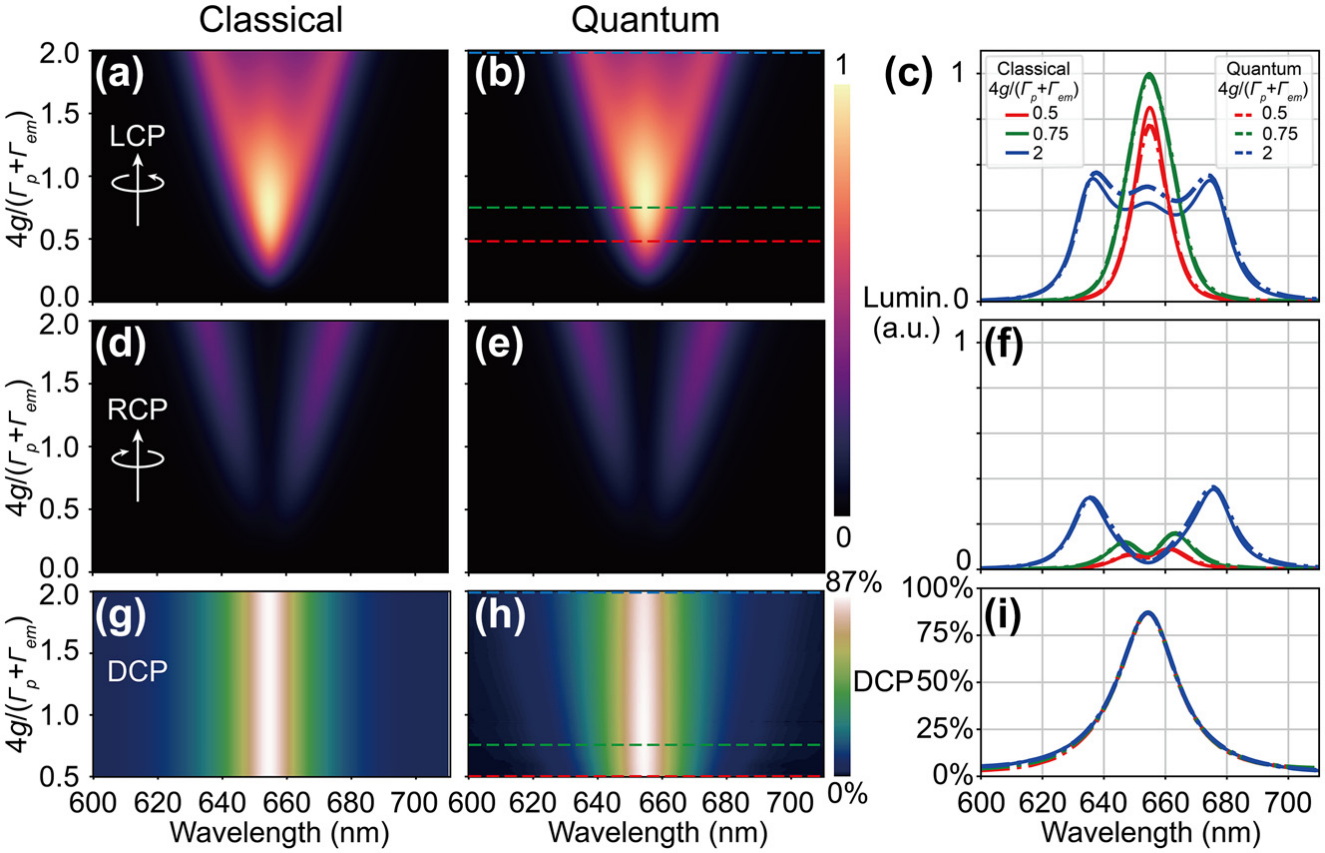
Modulation of luminescence DCP by the plasmon-emitter coupling predicted by classical and quantum models. (a)–(f) Luminescence mapping with respect to wavelength and coupling strength for LCP and RCP emissions based on chiral-COM and chiral-JCM. (c) and (f) show characteristic luminescence spectra for three different coupling strengths. (g)–(i) Luminescence DCP predicted by different models.

The excitation of *x* and *y* plasmon modes leads to significantly enhanced emitter luminescence, and their relative phase determines the emission chirality. Figure 6(a)–(f) show the luminescence spectra for LCP and RCP emission at different coupling strengths. Again, the chiral-COM/JCM gives very similar results and verify each other. It can be observed that the LCP luminescence is notably stronger than that of RCP, which is opposite to the CDS case. For LCP, the luminescence maximizes at intermediate coupling at the emitter wavelength 655 nm. Further increasing the coupling strength causes the spectrum to split into 3 branches and decrease in magnitude. On the contrary, the RCP luminescence remains dark at the emitter wavelength with only 2 branches, and its magnitude increases monotonically with coupling strength. Figure 6(g)–(i) show the luminescence DCP spectra. Interestingly, the value of DCP remains unchanged at around 87 % for all coupling strengths. This independency of the DCP on the coupling strength *g* can be intuitively explained by the COM. The chiral luminescence DCP is solely decided by the relative magnitudes and phases of the two orthogonal oscillating dipoles *μ*_*x*_ and *μ*_*y*_, whereas for pure circularly-polarized luminescence *μ*_*x*_ = ±*iμ*_*y*_. Therefore, the complex ratio *β* between *μ*_*x*_ and *μ*_*y*_ can serve as a figure of merit for chiral luminescence DCP. In Supplementary Material S21, we derive an analytical formula for *β* and DCP:

(8) $$\begin{array}{c} \beta =\frac{\mu_{y}\left(\omega \right)}{\mu_{x}\left(\omega \right)}=\frac{g_{y}\left(\omega_{x}^{2}-\omega^{2}-i\Gamma_{x}\omega \right)}{g_{x}\left(\omega_{y}^{2}-\omega^{2}-i\Gamma_{y}\omega \right)} \end{array}$$

(9) $$\begin{array}{c} DCP=\frac{{\left|\mu_{x}-i\mu_{y}\right|}^{2}-{\left|\mu_{x}+i\mu_{y}\right|}^{2}}{{\left|\mu_{x}-i\mu_{y}\right|}^{2}+{\left|\mu_{x}+i\mu_{y}\right|}^{2}}=\frac{2Imag\left(\beta \right)}{1+{\left|\beta \right|}^{2}} \end{array}$$

It is clear that *β* and thereby DCP only depend on *g*_*y*_/*g*_*x*_ and the magnitude of *g* cancels out in the division. The DCP value is also consistent with the circular differential local electric field at the emitter position (Figure 1(g)) as well as the DCP of radiative LDOS enhancement predicted by numerical simulations (Supplementary Material S18). It is worth mentioning that simulation results also indicate luminescence enhancement factors over 8000/2000 for LCP/RCP emissions, making the NBoM-emitter hybrid structure a highly pure, robust, and efficient chiral photon source.

Notably, the scattering and luminescence DCP show significantly different dependencies on the coupling strength, revealing two different physical mechanisms for the generation of chiral photons: (i) For the coupling-strength-dependent chiral scattering, the intrinsically-achiral NBoM cavity has mirror symmetry, showing identical far-field responses for LCP or RCP excitation (Figure 1(c)). The presence of the emitter breaks such symmetry, resulting in an intrinsic chiral response in the scattering of the NBoM-emitter hybrid. Since the chirality originates from the plasmon-emitter coupling, it is reasonable to expect that the CDS and scattering DCP would both depend on the plasmon-emitter coupling strength. (ii) For the coupling-strength-independent chiral luminescence, although the bare NBoM is achiral, it possesses a strong local extrinsic chirality as shown in Figure 1(d)–(g), as was also reported in previous literatures [15]. This local chirality is manifested by different radiative LDOS for LCP and RCP luminescence, with the DCP being solely dependent on the nanocavity design and emitter position, not on the coupling strength.

It is worth mentioning that the theoretical methods and main conclusions developed here are general and also applicable to other plasmonic nanocavity-emitter coupled systems (Supplementary Material S22). Thus, the proposed theoretical formalisms provide comprehensive understanding of the intriguing chiroptical responses of plasmonic nanocavity-emitter hybrid systems in terms of the CDS, chiral scattering, and chiral luminescence. Our results unveil their contrasted properties and distinct mechanisms, providing specific routes for engineering the chiroptical effects:

(i) The CDS, which is an important chiroptical effect in chiral sensing [67], [68], [69], [70], can be obtained by bridging two originally uncoupled, orthogonal plasmon eigenmodes with a third emitter, similar to the results reported in [71]. By carefully designing the energies and phases of the two plasmon modes, their destructive/constructive interference through the emitter can lead to different optical responses under LCP/RCP excitations. Note that, without this emitter as a bridge, there would be no CDS. Moreover, we show that the largest CDS can be obtained at intermediate coupling when $4g/\left(\Gamma_{p}+\Gamma_{em}\right)∼0.8$ , while further increase of the coupling strength causes splitting in the CDS spectra and reduction of its magnitude. The proper engineering of the coupling strength into the intermediate coupling regime is crucial for experimental nanophotonic devices with high CDS responses for sensing applications.
(ii) The same interference mechanism can also lead to chiral scattering under linearly-polarized excitation (i.e., linear-to-circular polarization conversion). Since two near-degenerated plasmon eigenmodes are coupled to the emitter, the excitation of either plasmon mode can drive the other orthogonal mode through the emitter with a tunable phase difference, leading to chiral scattering with prominent DCP. We show here that the scattering DCP can be either positive or negative at different wavelength regimes with its magnitude increasing with the coupling strength, which can potentially serve as an efficient and compact nanoscale platform for linear-to-circular polarization conversion. More importantly, our results demonstrate that the emitter types (dipole moment, damping rate, and orientation) and coupling strategies (nanocavity vacuum electric field, emitter position, and coupling strength) in the chiral scattering devices need to be carefully designed to reach the optimal coupling configuration and relative phases for desired device chiroptical properties.
(iii) The large extrinsic chirality of the nanocavities can be utilized to achieve high-purity chiral luminescence, with the DCP as high as 87 % shown in this work. The luminescence DCP is independent of the coupling strength and solely relies on the local extrinsic chirality. This leads to two direct design rules for chiral photon sources: (1) To achieve a high DCP in chiral luminescence, the plasmonic nanocavity needs to be designed with a large extrinsic chirality, and the emitter needs to be positioned within the optimal region in the plasmonic nanocavity (Figure 1(f)). (2) Since the luminescence DCP does not depend on the coupling strength, the purity of the chiral photons is robust in various coupling systems from Purcell-enhanced chiral luminescence to coherent chiral Rabi hybrid states. This result is particularly valuable for coupled systems where the quantum emitters have random dipole orientations.

Finally, the specific NBoM nanocavity proposed here has clear advantages over other plasmonic nanostructures for the realization of chiral photon devices: (1) The NBoM antenna belongs to the broad nanoparticle-on-mirror family [45], where the gap between the nanoparticle and the metallic mirror can easily reach down to several nanometers. The gap plasmon mode can create extremely large enhancement factors of the radiative LDOS for emitters located within the nanogap [3]. For our specific NBoM antenna, with a high emission DCP of 87 %, the enhancement factor of the radiative LDOS for the dominant emission handedness is over 8000 (Supplementary Material S18), which largely exceeds the enhancement factors of other common plasmonic nanostructures. (2) The large electric field enhancement as well as the large vacuum field in the nanogap for the NBoM antenna also creates the necessary condition for strong coupling between plasmon and single-emitter modes, making it interesting to explore the chiral degree of freedom for this nanogap system. The capability to adjust the coupling strength to enter the strong coupling regime grants the NBoM antenna a crucial degree of flexibility in controlling its chiroptical response. (3) As shown in the Supplementary Material S19, the angular distribution of the far-field emission energy of the dominant handedness light is majorly vertical relative to the substrate. Further, the emission DCP is almost constant and remains at a high level across a wide range of emission directions. These advantages make the NBoM antenna an ideal platform for the realization of ultra-bright, tunable, and directional on-chip chiral photon sources.

## 3 Conclusions

In summary, we extended the coupled oscillator and Jaynes–Cummings models to their chiral fashion and applied full-wave numerical simulations to study the chiral photon generation from a plasmonic nanocavity-emitter hybrid system, where the various manipulation methodologies such as nanocavity design, emitter type, and coupling strategy can be unified within a single theoretical framework. We show in a comprehensive manner how the interaction between plasmon modes and an achiral emitter leads to intriguing chiroptical responses in terms of CDS, chiral scattering, and chiral luminescence. Results reveal two distinct mechanisms for the generation of chiral photons in scattering and luminescence. The CDS and scattering DCP result from the intrinsic chirality generated by breaking the nanocavity mirror symmetry with the emitter, and thereby highly tunable by the coupling strength. Physically, the two originally-uncoupled, orthogonal plasmon modes interfere with each other through the emitter, leading to the chiroptical responses. On the other hand, the luminescence DCP relies on the extrinsic chirality of the nanocavity, independent of the coupling strength. Furthermore, the large extrinsic chirality of the proposed NBoM antenna (87 %) and its substantial enhancement of radiative LDOS (>8000) make the NBoM-emitter hybrid system a high-purity, robust and efficient chiral photon generator. The generalized theoretical approaches and analytical solutions developed in this study are supposed to serve as valuable guidelines for investigating and manipulating fundamental chiroptical phenomena.

## Supplementary Material

Supplementary Material Details
