# Supplementary material for: Manipulating chiral photon generation from plasmonic nanocavity-emitter hybrid systems: from weak to strong coupling
Source: Nanophotonics. 2024 Jan 16;13(3):357–68. doi: 10.1515/nanoph-2023-0738 (PMC11501219; doi:10.1515/nanoph-2023-0738)
Supplement: Supplementary file 1 — Supplementary Material Details [file j_nanoph-2023-0738_suppl_001.docx]

**Manipulating Chiral Photon Generation from Plasmonic Nanocavity-Emitter Hybrid Systems: from Weak to Strong Coupling**

Jian Yang^1^, Huatian Hu^2,3,*^, Qingfeng Zhang^4^, Shuai Zu^5^, Wen Chen^1^, and Hongxing Xu^1,6,*^

^1^ State Key Laboratory of Precision Spectroscopy, School of Physics and Electronic Science, East China Normal University, Shanghai 200241, China

^2^ Hubei Key Laboratory of Optical Information and Pattern Recognition, Wuhan Institute of Technology, Wuhan 430205, China

^3^ Center for Biomolecular Nanotechnologies, Istituto Italiano di Tecnologia, Via Barsanti 14, 73010 Arnesano (LE), Italy

*^4^* *College of Chemistry and Molecular Sciences, Wuhan University, Wuhan 430072, China*

*^5^ Advanced Photonics Center, School of Electronic Science and Engineering, Southeast University, Nanjing 210096, China*

^6^ The Institute of Advanced Studies, School of Physics and Technology, Center for Nanoscience and Nanotechnology, and Key Laboratory of Artificial Micro- and Nano-structures of Ministry of Education, Wuhan University, Wuhan 430072, China

^*^ Corresponding authors: huatian.hu@iit.it and [hxxu@whu.edu.cn](mailto:hxxu@whu.edu.cn)

[S1 Finite element simulations 3](#_Toc153555175)

[S2 Classical chiral-COM 3](#_Toc153555176)

[S3 Quantum chiral-JCM 3](#_Toc153555177)

[S4 Dependence of extrinsic chirality on nanobar shape 4](#_Toc153555178)

[S5 System tolerance to deviation in geometry 5](#_Toc153555179)

[S6 Angular distribution of CDS 6](#_Toc153555180)

[S7 Origin of the extrinsic chirality 8](#_Toc153555181)

[S8 Extrinsic chirality and optical chirality 9](#_Toc153555182)

[S9 Breaking the mirror symmetry of the NBoM with the emitter 10](#_Toc153555183)

[S11 Simulated extinction and absorption spectra for the NBoM-emitter hybrid structure 11](#_Toc153555184)

[S12 Effect of the cross-coupling terms on scattering for chiral-COM 12](#_Toc153555185)

[S13 Deduction of the CDS for chiral-COM 13](#_Toc153555186)

[S14 Discrepancy in scattering spectra between numerical simulations and theoretical models 14](#_Toc153555187)

[S15 Extinction spectra calculated by chiral-COM 14](#_Toc153555188)

[S16 Manipulating CDS by changing the gap property 15](#_Toc153555189)

[S17 Angular distribution of chiral scattering DCP 16](#_Toc153555190)

[S18 Simulated radiative LDOS for LCP/RCP light 17](#_Toc153555191)

[S19 Angular distribution of chiral luminescence DCP 18](#_Toc153555192)

[S20 Evolution of the oscillator dipole moments for chiral-COM 18](#_Toc153555193)

[S21 Independency of the luminescence DCP on the coupling strength 19](#_Toc153555194)

[S22 Application of the theoretical model to other plasmonic nanocavity-emitter systems 19](#_Toc153555195)

[References 21](#_Toc153555196)

S1 **Finite element simulations**

Numerical simulations were performed with COMSOL Multiphysics based on the finite element method. An *x*-*y* degeneracy-lifted 62 nm long, 60 nm wide, 60 nm thick silver nanobar whose corners were rounded by a curvature of 8 nm was placed on a semi-infinite silver substrate spaced by a 5 nm thick dielectric spacer. Perfectly matched layers were implemented at the simulation domain boundaries to absorb outgoing electromagnetic waves and prevent reflection. For simulating the scattering from the nanocavity-emitter hybrid structure, a port at the top boundary was used as a plane-wave input for illuminating the nanostructure. The emitter was implemented as a small nanocylinder with radius of 2 nm and thickness of 3 nm at the corner of the nanobar embedded within the spacer layer. The permittivity of the emitter was modeled using the Lorentzian dielectric function: $\epsilon_{em}\left( \omega\right)=\epsilon_{\infty}-f\omega_{em}^{2}/(\omega^{2}-\omega_{em}^{2}+i\Gamma_{em}\omega)$ where $\epsilon_{\infty}=2.25$, $\omega_{em}=1.89 eV$ and $\Gamma_{em}=25 meV$. The oscillator strength *f* can tune the plasmon-emitter coupling strength. For luminescence simulations, an electric point dipole oscillating along the *z* direction (direction with the largest field enhancement) at the nanocylinder position replaced the port as the excitation source. Near-to-far-field transformation [1] was performed to obtain the far-field scattering and luminescence power for left-/right-handed-circular-polarization (LCP/RCP), respectively. The permittivity of silver was interpolated based on tabulated measured data [2], and the refractive index of the dielectric spacer was set to 1.5.

S2 Classical chiral-COM

For the classical chiral-COM, the *x*, *y* plasmon modes and the emitter were treated as three coupled harmonic oscillators with their equations of motion fully described by the main text Equations (1)-(3). *(i) For the scattering problems*, under monochromatic driving, the three oscillators would oscillate in the same frequency as the driving force, so the dipole moments follow $\mu_{x,y,em}=\tilde{A}_{x,y,em}e^{-i\omega t}$ and the sets of differential equations turn into algebraic equations which were solved analytically. Since the scattering cross-section of the emitter is much smaller than that of the plasmonic nanocavity, the total scattering of the hybrid structure can be calculated by $Q_{scat}\propto\omega^{4}(\left| \mu_{x} \right|^{2}+\left| \mu_{y} \right|^{2})$. The extinction of the hybrid structure can be calculated as the time-averaged work done by the external driving force: $Q_{ext}\propto\left\langle F_{x}\dot{\mu}_{x}+F_{y}\dot{\mu}_{y} \right\rangle$ [3]. *(ii) For the luminescence problems,* we removed the external forces $F_{x}=F_{y}=0$ and set the emitter to be initially populated $\mu_{em}\left( 0 \right)=1$ in main text Equations (1)-(3). Then we obtained the time evolution of the three dipole moments by solving Equations (1)-(3) numerically with the open-source python SciPy package. The luminescence spectra for LCP and RCP (main text Figure 6) can be calculated by first forming LCP/RCP dipole moments $\mu_{\pm}=\mu_{x}\pm i\mu_{y}$ and then taking the Fourier transform of their radiation power.

S3 Quantum chiral-JCM

The quantum chiral-JCM was implemented with the open-source python QuTiP package [4,5]. *(i) For the scattering problems*, the system Hamiltonian is shown in the main text Equation (7). Damping was taken into account using the collapse operators $\sqrt{\Gamma_{x}}a_{x}$, $\sqrt{\Gamma_{y}}a_{y}$ and $\sqrt{\Gamma_{em}}\sigma_{-}$ based on the Lindblad master equation. Under steady state, the far-field scattering is proportional to the boson number of the cavity and further corrected for the fourth-power frequency dependence for dipole emission approximation: $Q_{scat}\propto\omega^{4}(\left\langle a_{x}^{\dagger}a_{x} \right\rangle+\left\langle a_{y}^{\dagger}a_{x} \right\rangle)$. *(ii) For the luminescence problems*, the Hamiltonian of the system is as follows:

$$\begin{aligned} H=\omega_{x}a_{x}^{\dagger}a_{x}+\omega_{y}a_{y}^{\dagger}a_{y}+\omega_{em}\sigma_{+}\sigma_{-}+g_{x}\left( a_{x}^{\dagger}\sigma_{-}+a_{x}\sigma_{+} \right)+g_{y}\left( a_{y}^{\dagger}\sigma_{-}+a_{y}\sigma_{+} \right) \#(S1) \end{aligned}$$

The emitter has an initial population of one while the cavity modes are at zero. This initial value does not affect the final results due to the normalization. The time evolution of the populations can be obtained by solving for the system states numerically within the QuTiP package. To calculate the luminescence spectra, a constant small pumping continuously populates the emitter energy level. Steady-state correlation functions of the LCP/RCP operators $a_{\pm}=a_{x}\pm ia_{y}$ were calculated and then the Fourier transform was performed.

S4 Dependence of extrinsic chirality on nanobar shape

To obtain the optimal extrinsic chirality of the bare nanobar-on-mirror (NBoM) cavity, we varied the nanobar shape by changing its dimension along the *y* axis. Figure S1 shows the scattering spectra and electric field distribution for $L_{y}$ values from 60 to 65 nm. When $L_{y}=L_{x}=60 nm$, the plasmon modes along the *x* and *y* axes are degenerate and there is only one peak in the scattering spectrum. The electric field distribution has a four-fold rotational symmetry. When we increase $L_{y}$, the mode along *y* axis redshifts and the degeneracy is lifted. In the scattering spectra, the two modes are separated with different extents of overlap decided by the difference between $L_{x}$ and $L_{y}$. The electric field distribution only has a two-fold rotational symmetry. It can be seen that the short-wavelength mode corresponds to the cavity *x* mode and the long-wavelength mode corresponds to the cavity *y* mode. Note that the scattering spectra are identical for LCP and RCP excitations for the bare NBoM. The field distributions are for LCP excitations, while those for RCP excitations are along the opposite diagonal (main text Figure 1(c) and (d)). Therefore, only local extrinsic chirality exists and there is no far-field chirality for the bare NBoM.


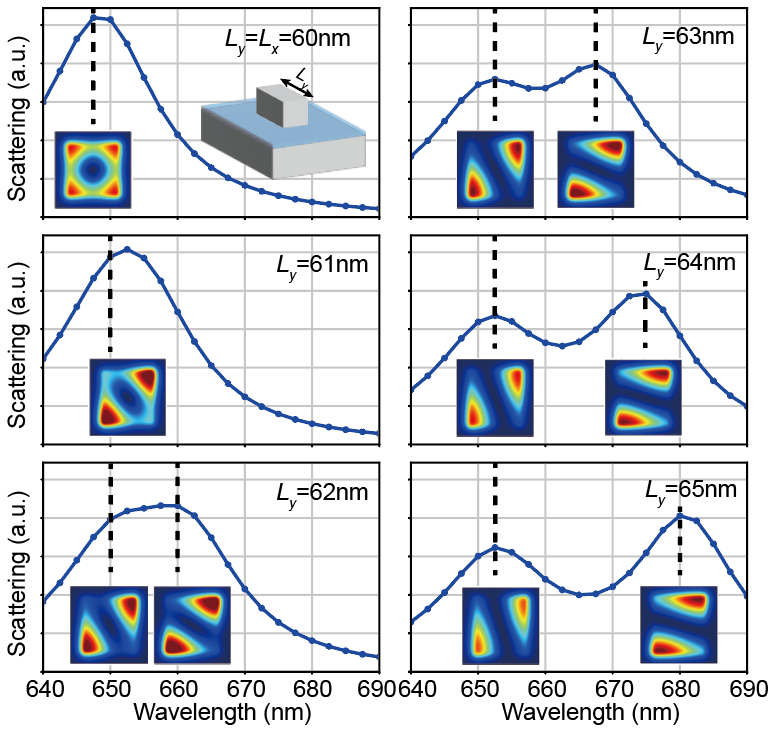


**Figure S1:** Scattering spectra of the bare NBoM with different $L_{y}$. The insets show the ${|\boldsymbol{E}/\boldsymbol{E}_{\boldsymbol{0}}|}^{2}$ distributions at the central plane of the nanogap for LCP excitation.

Figure S2 shows the maximum ${|\boldsymbol{E}/\boldsymbol{E}_{\boldsymbol{0}}|}^{2}$ (left axis) and corresponding $\rho_{CP}$ (right axis) for the bare NBoM with different $L_{y}$. Interestingly, both ${|\boldsymbol{E}/\boldsymbol{E}_{\boldsymbol{0}}|}^{2}$ and $\rho_{CP}$ are maximized for $L_{y}=62 nm$. Possible reasons are as follows. When $L_{x}$ and $L_{y}$ are too close, the two cavity modes are almost degenerate and have a phase difference near $\pi/2$. Therefore, the interference is neither constructive nor destructive, leading to moderate field enhancement and a small $\rho_{CP}$. However, when $L_{x}$ and $L_{y}$ are too far away, although two modes may have phase difference near 0 or $\pi$ for LCP or RCP excitations and interfere constructively or destructively, the mode energies become too much separated and do not overlap. Only with a tiny length mismatch of 2 nm can the system achieve an optimal extrinsic chirality. Therefore, we choose $L_{y}=62 nm$ for the whole main text and the remaining of the Supplementary Material.


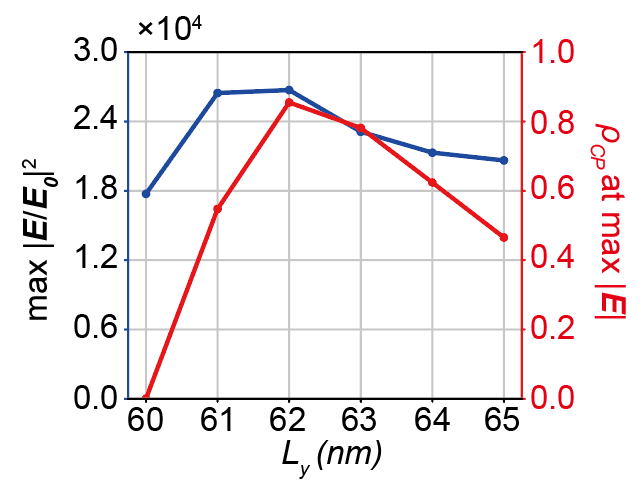


**Figure S2:** Maximum ${|\boldsymbol{E}/\boldsymbol{E}_{\boldsymbol{0}}|}^{2}$ (left axis) and corresponding $\rho_{CP}$ (right axis) for the bare NBoM with different $L_{y}$.

S5 System tolerance to deviation in geometry

Despite the great advance in nanofabrication techniques, the precise control of the nanocavity size and quantum emitter position down to one or two nanometers is still challenging. Duan et al. demonstrated nanofabrication with precision down to sub-nanometer scale using electron beam lithography [6,7]. Nevertheless, we propose that our system has relatively large tolerances to the nanocavity size and quantum emitter position.

**Tolerance of the size in nanofabrication.** For our optimal design, the size of the nanobar is 60 × 62 × 60 nm^3^. It exhibits an exceptionally large circular differential electric field $\rho_{CP}=(\left| \boldsymbol{E}_{\boldsymbol{L}} \right|^{2}-\left| \boldsymbol{E}_{\boldsymbol{R}} \right|^{2})/(\left| \boldsymbol{E}_{\boldsymbol{L}} \right|^{2}+\left| \boldsymbol{E}_{\boldsymbol{R}} \right|^{2})$ of 87% and a simultaneous large field enhancement $\left| \boldsymbol{E}_{\boldsymbol{L}}\boldsymbol{/}\boldsymbol{E}_{\boldsymbol{0}} \right|^{2}$ of $2.6\times{10}^{4}$ which takes advantage of the nanoparticle-on-mirror systems. Due to the principle of reciprocity, the square of the electric field enhancement can directly reflect the LDOS enhancement. To test the system tolerance to size deviation in realistic nanofabrication, we changed the nanobar length along the *y* direction and calculated the achievable $\rho_{CP}$ and $\left| \boldsymbol{E}_{\boldsymbol{L}}\boldsymbol{/}\boldsymbol{E}_{\boldsymbol{0}} \right|^{2}$ values. Since the positions with the maximal $\rho_{CP}$ and the maximal $\left| \boldsymbol{E}_{\boldsymbol{L}}\boldsymbol{/}\boldsymbol{E}_{\boldsymbol{0}} \right|^{2}$ do not exactly coincide with each other, we picked regions with $\rho_{CP}$ larger than certain levels and investigated the achievable maximal $\left| \boldsymbol{E}_{\boldsymbol{L}}\boldsymbol{/}\boldsymbol{E}_{\boldsymbol{0}} \right|^{2}$. As shown in Figure S3, for all three $\rho_{CP}$ levels (40%, 60%, and 80%), the maximal $\left| \boldsymbol{E}_{\boldsymbol{L}}\boldsymbol{/}\boldsymbol{E}_{\boldsymbol{0}} \right|^{2}$ reduces with increasing $L_{y}$ as expected. Nevertheless, for a nanobar of 60 × 66 × 60 nm^3^, $\rho_{CP}\geq80\%$ and $\left| \boldsymbol{E}_{\boldsymbol{L}}\boldsymbol{/}\boldsymbol{E}_{\boldsymbol{0}} \right|^{2}\geq{10}^{4}$ can be obtained simultaneously. Even for a nanobar of 60 × 80 × 60 nm^3^, simultaneous $\rho_{CP}\geq80\%$ and $\left| \boldsymbol{E}_{\boldsymbol{L}}\boldsymbol{/}\boldsymbol{E}_{\boldsymbol{0}} \right|^{2}\geq{10}^{3}$ can be easily obtained. This clearly demonstrates that our system is robust to the deviation in nanobar size.


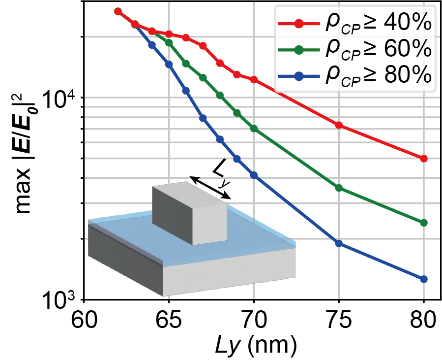


**Figure S3:** System tolerance to the deviation in the nanobar size.

**Tolerance of the deviation of the quantum emitter.** Next, we show that our system is also robust to the deviation in the location of the quantum emitter. As shown in Figure S4, we calculated the $\rho_{CP}$ and $\left| \boldsymbol{E}_{\boldsymbol{L}}\boldsymbol{/}\boldsymbol{E}_{\boldsymbol{0}} \right|^{2}$ when the quantum emitter deviates from the optimal position indicated in Figure 1(f). Again, the square of the electric field enhancement can directly reflect the LDOS enhancement based on the principle of reciprocity. Since the deviation direction is random in experiments, we averaged the results for 4 points along the $\pm x$ and $\pm y$ directions (inset in Figure S4). The $\rho_{CP}$ is robust against changes in the emitter position and remains above 80% even for a 10 nm deviation. On the other hand, the $\left| \boldsymbol{E}_{\boldsymbol{L}}\boldsymbol{/}\boldsymbol{E}_{\boldsymbol{0}} \right|^{2}$ decreases with the deviation. Nevertheless, it remains above $2\times{10}^{4}$ for a 5 nm deviation, and above ${10}^{4}$ for a 10 nm deviation. Therefore, our system also has a large tolerance to the deviation in the position of the quantum emitter.


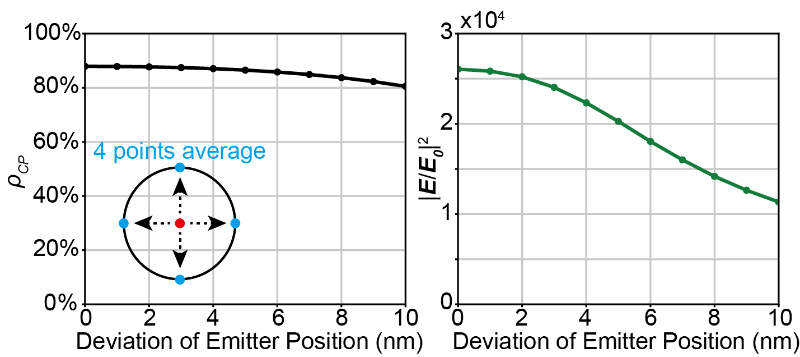


**Figure S4:** System tolerance to the deviation in the position of the quantum emitter.

Finally, we want to briefly discuss about possible rotation of the emitter dipole orientation. At the emitter position in our NBoM nanocavity, the dominant electric field vector component is along the *z* axis. Therefore, the plasmon mode only effectively couples with the *z* component of the emitter dipole. Quantum emitters like molecules and transition metal dichalcogenides have well-defined dipole orientations. Depending on the nanofabrication technique, the orientation of these emitters’ dipoles may not be easily controllable. Nevertheless, the rotation of the emitter dipole orientation is equivalently changing the *z* component of the dipole moment, effectively leading to different coupling strengths $g$. It is worth mentioning that the independence of the chiral luminescence DCP on coupling strength (and thereby emitter dipole orientation) is beneficial for the realization of robust chiral photonic sources.

S6 Angular distribution of CDS

We analyzed the angular distribution of the far-field scattering power density under LCP and RCP excitations, respectively. As shown by the polar plots of Figure S5(a), for a 60 × 60 × 60 nm^3^ nanocube, the far-field scattering shows an azimuthal-angle independent distribution as a circular shape, with no differences between LCP and RCP excitations. When we lift the degeneracy between *x* and *y* directions by changing the nanocube to a nanobar with a size of 60 × 62 × 60 nm^3^, the scattering angular distribution becomes dependent on the azimuthal angle with an elliptical shape in the polar plot (Figure S5(b)). Note that the major axis of the elliptical distribution is along 45^o^(225^o^) azimuthal direction for LCP excitation and 135^o^(315^o^) for RCP excitation. Therefore, although the overall integral scattering shows no CDS (main text Figure 1(c)), the angular distribution of CDS is nonzero and reaches a magnitude of 0.75 at oblique collection angles along the 45^o^, 135^o^, 225^o^ and 315^o^ azimuthal directions. This is another manifestation of the nanobar extrinsic chirality by breaking the mirror symmetry of the system through the collection angle. For collection angles along the 0^o^ and 180^o^ azimuthal directions, the mirror symmetry still exists, leading to zero CDS.


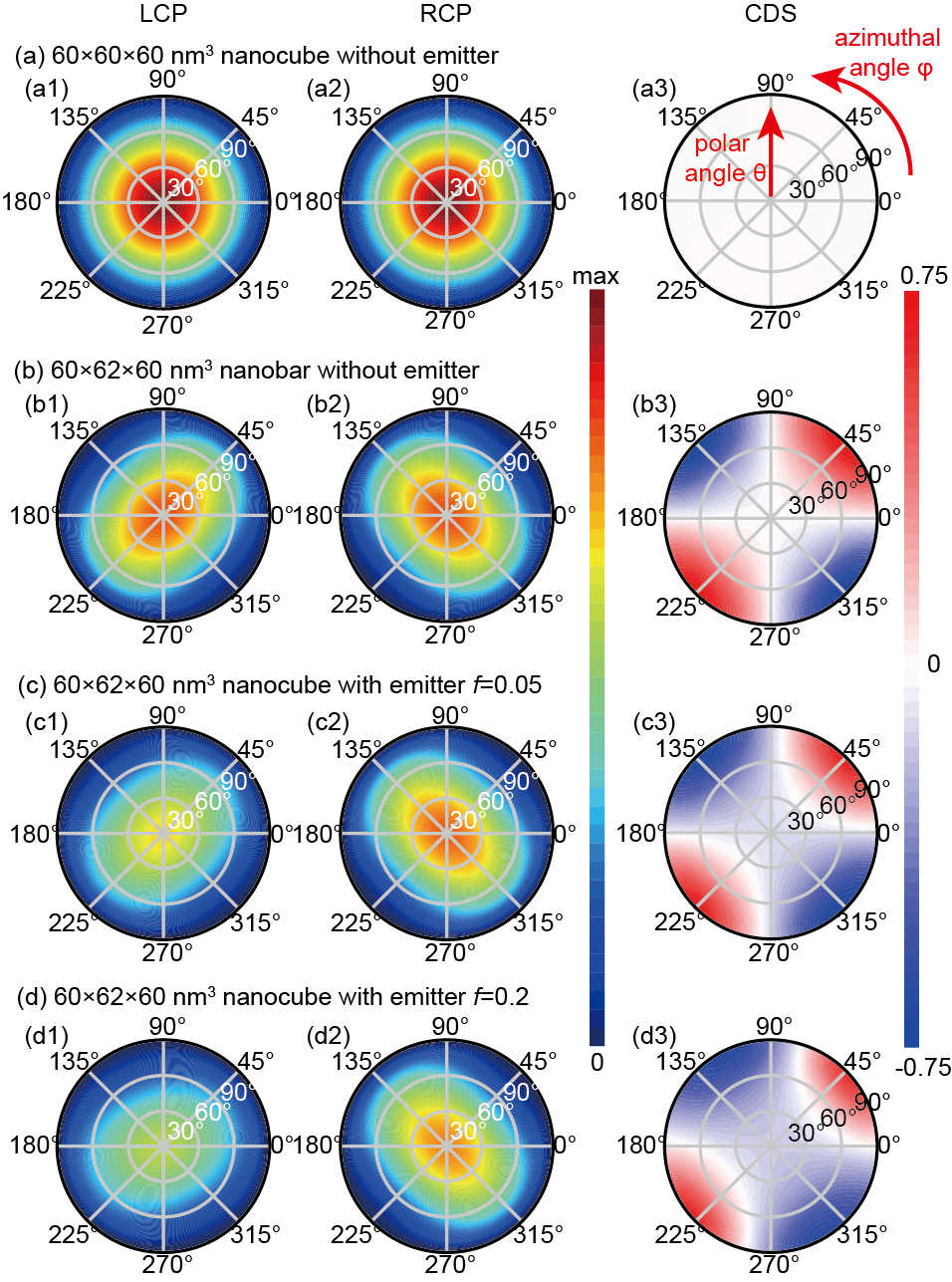


**Figure S5**: Angular distribution of CDS for (a) bare nanocube without degeneracy lifting, (b) bare nanobar with degeneracy lifting, (c) and (d) nanobar coupled with an emitter with oscillator strength (c) *f*=0.05 and (d) *f*=0.2. Here, in the polar plots, the radial axis corresponds to the polar angle, the angular axis corresponds to the azimuthal angle, and the colormap represents the intensity distribution. The CDS mapping shows the circular differential scattering between LCP and RCP excitations along different directions.

As shown in the main text Figure 2, when a quantum emitter was introduced in the nanogap at the nanobar corner, the mirror symmetry of the system was broken and nonzero CDS was observed in the integral scattering spectra. In Figure S5(c), we calculated the angular distribution of far-field scattering for the coupled system under LCP and RCP excitations. When the quantum emitter coupled with the nanobar, the elliptical distribution of the scattering power density persevered. However, the scattering intensity under LCP excitation was noticeably smaller than that for RCP excitation. This leads to a dominant negative CDS distribution, manifested as larger regions with negative CDS in the CDS mapping (Figure S5(c3)). Furthermore, the region with negative CDS grows with the increase of the emitter oscillator strength *f*, indicating more negative CDS for larger coupling strength between the plasmonic nanocavity and the quantum emitter (Figure S5(d)). This is in agreement with the integral CDS behavior presented in the main text Figure 2.

S7 Origin of the extrinsic chirality

The opposite diagonal distribution of ${|\boldsymbol{E}/\boldsymbol{E}_{\boldsymbol{0}}|}^{2}$ for LCP and RCP excitations can be explained by a simple harmonic oscillator model. In the bare NBoM, the cavity modes along the *x* and *y* directions can be described by two uncoupled simple harmonic oscillators:

$$\begin{aligned} \ddot{\mu}_{x}+\Gamma_{x}\dot{\mu}_{x}+\omega_{x}^{2}\mu_{x}=F_{x}\#(S1) \end{aligned}$$

$$\begin{aligned} \ddot{\mu}_{y}+\Gamma_{y}\dot{\mu}_{y}+\omega_{y}^{2}\mu_{x}=F_{y} \#\left( S2 \right) \end{aligned}$$

where $\mu_{x,y}$ are the electric dipole moments, $\omega_{x,y}$ are the mode angular frequencies (energies), and $\Gamma_{x,y}$ are the dissipation rates (FWHM) of the cavity *x* and *y* modes, respectively. $F_{x}=F_{0}exp(-i\omega t)$ and $F_{y}=F_{0}exp(-i\left( \omega t+\varphi_{F} \right))$ are the external driving fields along the *x* and *y* directions. $\varphi_{F}$ is the phase difference between *x* and *y* directions and is $\pm\pi/2$ for LCP and RCP excitations. Analytical formula for the electric dipole moments can be easily derived:

$$\begin{aligned} \mu_{x}\left( \omega\right)=F_{0}\frac{1}{\omega_{x}^{2}-\omega^{2}-i\Gamma_{x}\omega} \#\left( S3 \right) \end{aligned}$$

$$\begin{aligned} \mu_{y}\left( \omega\right)=F_{0}\frac{e^{-i\varphi_{F}}}{\omega_{y}^{2}-\omega^{2}-i\Gamma_{y}\omega} \#\left( S4 \right) \end{aligned}$$

We then define the phase difference between $\mu_{x}$ and $\mu_{y}$ as:

$$\begin{aligned} \varphi_{\mu}=-\left( \arg\left( \mu_{y} \right)-\arg\left( \mu_{x} \right) \right)\#\left( S5 \right) \end{aligned}$$

Figure S6 shows the dependence of $\varphi_{\mu}$ on $\varphi_{F}$ and wavelength $\lambda$. In the left panel of Figure S6, the excitation wavelength is fixed at 655 nm between the energy levels of two oscillators. When the incident polarization is LCP, as indicated by the blue dashed lines, the phase difference between the *x* and *y* oscillators is close to 0, so the overall electric field is distributed along the diagonal connecting the 1^st^ and 3^rd^ quarters (main text Figure 1(d)). When the incident polarization is RCP, as indicated by the green dashed lines, the phase difference is close to $\pi$, so the overall electric field is distributed along the diagonal connecting the 2^nd^ and 4^th^ quarters (main text Figure 1(e)). In the right panel of Figure S6, the excitation phase lag is fixed at $\pm\pi/2$ corresponding to LCP and RCP, respectively. Only when the excitation wavelength is between the energy levels of the two oscillators can we see a nontrivial $\varphi_{\mu}$ that is different from incident phase lag $\varphi_{F}$.


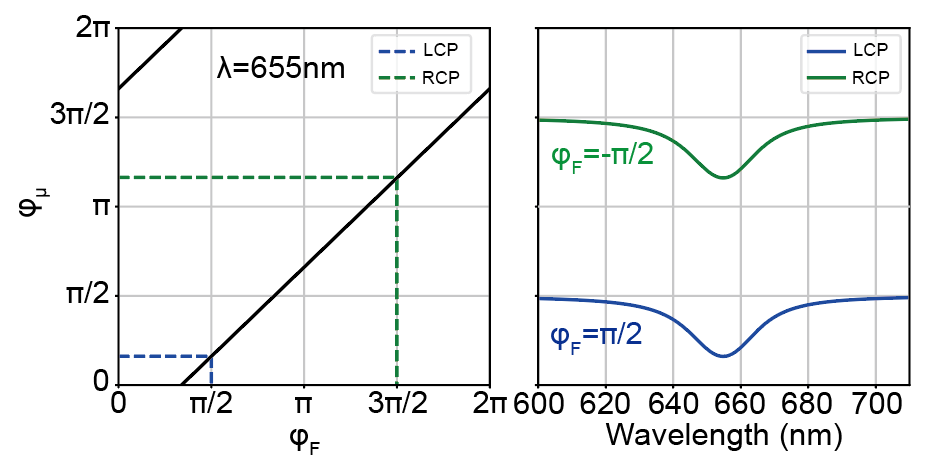


**Figure S6:** Phase difference between $\mu_{y}$ and $\mu_{x}$ for different excitation phase lag $\varphi_{F}$ and wavelength, calculated by the simple harmonic oscillator model.

S8 Extrinsic chirality and optical chirality

The extrinsic chirality characterizes the circular differential electric field distribution under LCP and RCP excitations. There is another important physical quantity, the optical chirality (OC), defined as $OC=-\frac{\epsilon_{0}\omega}{2}Im[\boldsymbol{E}^{*}\cdot\boldsymbol{B}]$. OC measures the chirality of local electric and magnetic fields, and can characterize the enhancement of circular dichroism in chiral molecule detection. It would be interesting to discuss the relation between the OC and extrinsic chirality.

Nevertheless, we do not see a direct connection between the OC and the extrinsic chirality. We have calculated the OC distribution within the nanogap for LCP and RCP excitations. As shown in Figure S7, for both LCP and RCP excitations, the 60 × 62 × 60 nm^3^ nanobar exhibits a four-lobe OC distribution within the nanogap, and the OC value approaches zero at the diagonals with the most prominent extrinsic chirality. Clearly the OC and circular differential electric field $\rho_{CP}$ (main text Figure 1(f)) have distinct distributions. Moreover, nonzero local OC even exists for a 60 × 60 × 60 nm^3^ nanocube which does not show extrinsic chirality from the scattering at all.


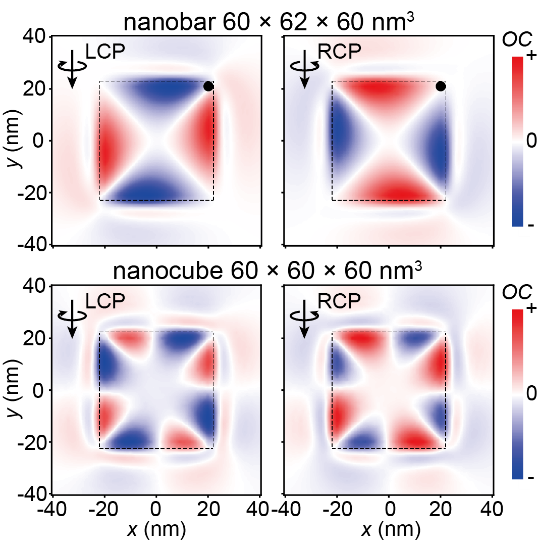


**Figure S7:** Optical chirality distribution within the nanogap for nanobar and nanocube.

We propose the following argument for the different behavior of OC and extrinsic chirality. OC measures the local chirality of the electric and magnetic fields, and is determined by the directions, magnitudes, and phases of the electric and magnetic field vectors. Large OC can be produced at certain regions near plasmonic nanostructures even with high symmetry (nanocubes). On the other hand, the extrinsic chirality we have been studying here is the circular differential electric field distribution, which measures the difference in the local electric field intensity between LCP and RCP excitations. It is not directly dependent on the magnetic field, and it only exists for degeneracy-lifted nanobars but not for nanocubes. Therefore, it appears that the OC and extrinsic chirality may not exhibit a direct correlation with each other.

S9 Breaking the mirror symmetry of the NBoM with the emitter

For scattering problems, the bare NBoM nanocavity has mirror symmetry (Figure S8(a)) and its scattering spectra show no circular differential scattering (CDS) (main text Figure 1(c)). When a quantum emitter is embedded within the nanogap at the corner of the nanobar (Figure S8(b)), the mirror symmetry of the system is broken due to the local modified permittivity. Then the NBoM-emitter hybrid system can have CDS signal (Figure S8(c) and main text Figures 2,3). It also generates chiral scattering under *x*-polarized excitation (main text Figure 4).

For luminescence problems, although the bare NBoM nanocavity has no intrinsic chirality, it has giant extrinsic chirality at the nanogap corners (main text Figure 1(d-g)). When an achiral quantum emitter is placed at the corner, it can utilize this local chirality to generate highly chiral and efficient luminescence (Figure S8(d) and main text Figure 6).


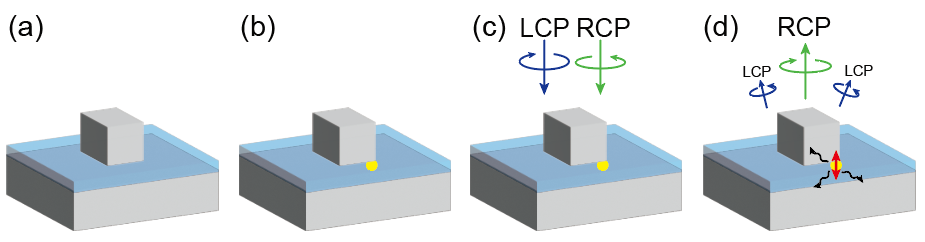


**Figure S8:** 3D Schematic showing the mirror symmetry breaking by the quantum emitter.

**S10 More simulated scattering spectra for the NBoM-emitter hybrid structur**e

Figure S9 shows more simulated scattering spectra for the NBoM-emitter hybrid structure for different emitter oscillator strengths. The modes splitting as well as CDS increases with the coupling strength. Since the CDS originates from the symmetry breaking induced by the emitter, it is reasonable to expect that larger oscillator strength values can induce stronger coupling and larger symmetry breaking, thereby leading to larger CDS magnitudes.


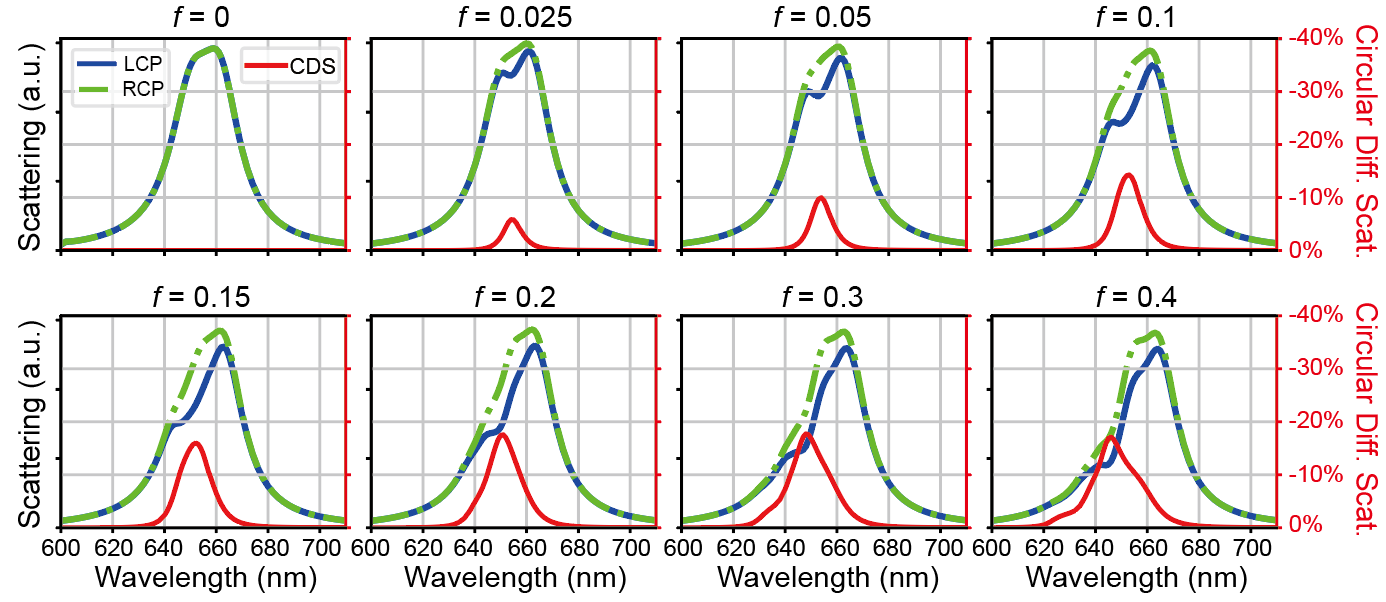


**Figure S9:** Simulated scattering and CDS spectra for the NBoM-emitter hybrid structure for different emitter oscillator strengths.

Figure S10 shows the dependence of CDS on the emitter size. With increasing emitter sizes, the symmetry breaking becomes larger, also leading to larger CDS magnitudes.


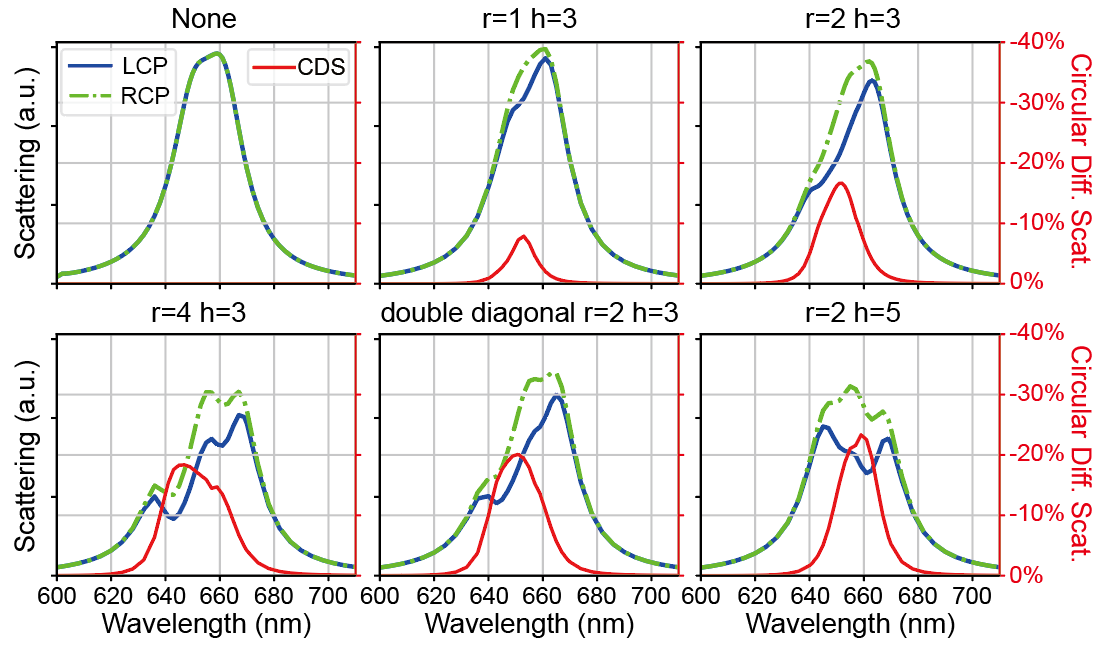


**Figure S10:** Simulated scattering and CDS spectra for the NBoM-emitter hybrid structure for different emitter sizes. The size unit is nm.

S11 Simulated extinction and absorption spectra for the NBoM-emitter hybrid structure

While far-field scattering spectra show a negative $\rho_{CP}$, the extinction spectra are identical for LCP and RCP, and the absorption spectra show a positive $\rho_{CP}$ (Figure S11). Apparently, the circular dichroism in scattering and absorption compensates each other, rendering the extinction insensitive to the incident polarization state.


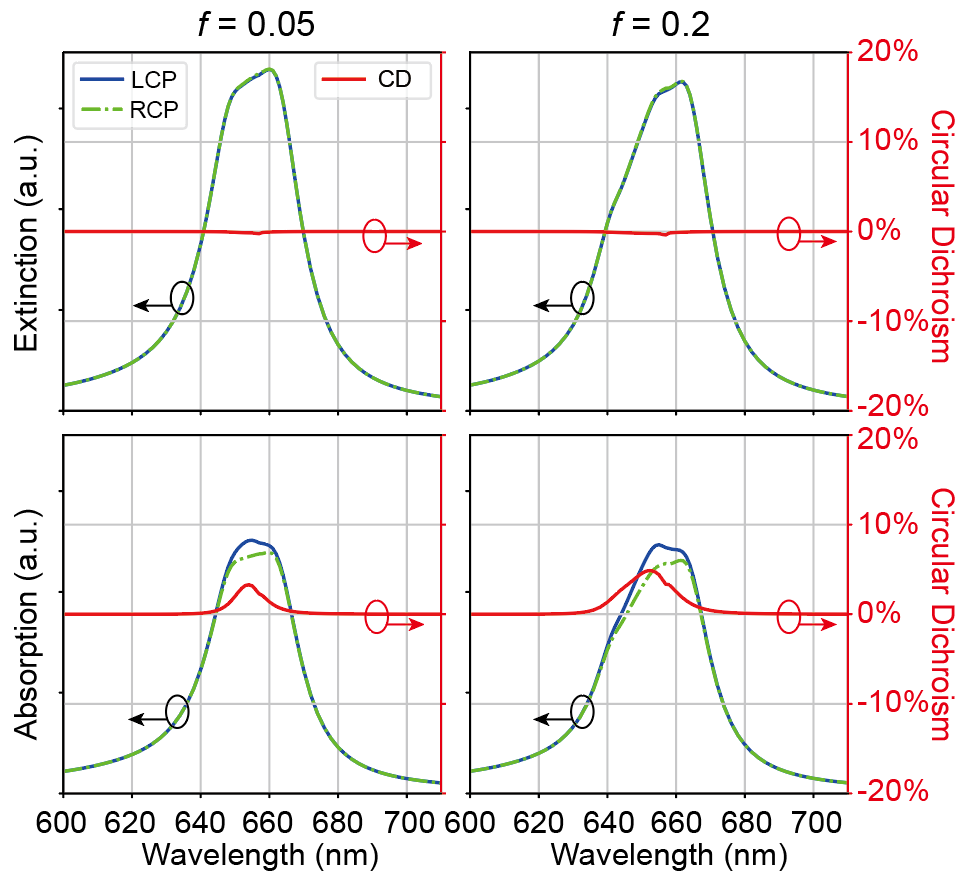


**Figure S11:** Simulated extinction, absorption and their CD spectra for the NBoM-emitter hybrid structure.

S12 Effect of the cross-coupling terms on scattering for chiral-COM

According to the main text Equation (4), the dipole moment of cavity *x* mode under different incident polarization states can be written as:

$$\begin{aligned} \mu_{x}\left( \omega, LCP \right)=F_{0}\frac{L_{y}\left( \omega\right)L_{em}\left( \omega\right)-{4\omega}^{2}g_{y}^{2}+4i\omega^{2}g_{x}g_{y}}{L_{x}\left( \omega\right)L_{y}\left( \omega\right)L_{em}\left( \omega\right)-{4L}_{x}\left( \omega\right)\omega^{2}g_{y}^{2}-4L_{y}\left( \omega\right)\omega^{2}g_{x}^{2}}\#\left( S6 \right) \end{aligned}$$

$$\begin{aligned} \mu_{x}\left( \omega, RCP \right)=F_{0}\frac{L_{y}\left( \omega\right)L_{em}\left( \omega\right)-{4\omega}^{2}g_{y}^{2}-4i\omega^{2}g_{x}g_{y}}{L_{x}\left( \omega\right)L_{y}\left( \omega\right)L_{em}\left( \omega\right)-{4L}_{x}\left( \omega\right)\omega^{2}g_{y}^{2}-4L_{y}\left( \omega\right)\omega^{2}g_{x}^{2}}\#\left( S7 \right) \end{aligned}$$

$$\begin{aligned} \mu_{x}\left( \omega, x-polar \right)=F_{0}\frac{L_{y}\left( \omega\right)L_{em}\left( \omega\right)-{4\omega}^{2}g_{y}^{2}}{L_{x}\left( \omega\right)L_{y}\left( \omega\right)L_{em}\left( \omega\right)-{4L}_{x}\left( \omega\right)\omega^{2}g_{y}^{2}-4L_{y}\left( \omega\right)\omega^{2}g_{x}^{2}}\#\left( S8 \right) \end{aligned}$$

The cross-coupling term $\pm4i\omega^{2}g_{x}g_{y}$ in the numerator comes from energy transfer from the cavity *y* mode through the emitter. The scattering spectra of $\mu_{x}$ is then $scat(\mu_{x})\propto\omega^{4}\left| \mu_{x} \right|^{2}$. The left panel of Figure S12 shows the scattering spectra of cavity *x* mode under different incident polarization states. The opposite-sign cross-coupling terms for LCP and RCP cause a significant difference in the scattering spectra. Similar effects can be observed for $\mu_{y}$ scattering (center panel) and total scattering (right panel).


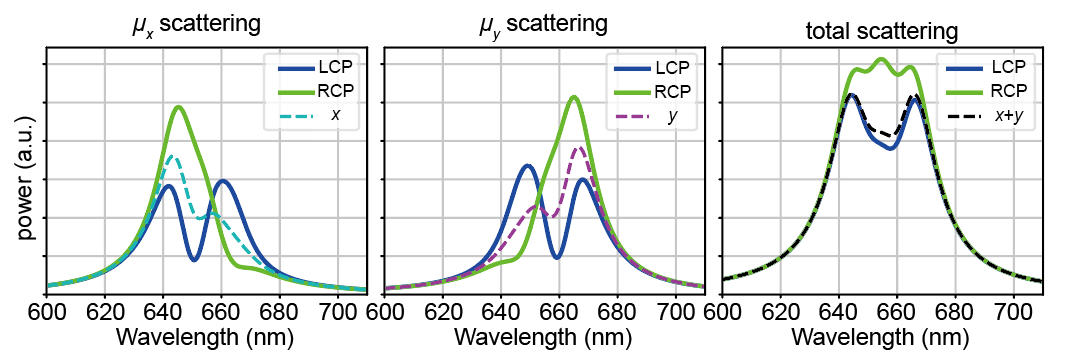


**Figure S12:** Effects of the cross-coupling terms in the COM analytical formula for dipole moments on the scattering spectra for normalized coupling strength $4g/(\Gamma_{p}+\Gamma_{em})=0.84$.

S13 Deduction of the CDS for chiral-COM

According to Equations (S6) and (S7):

$$Q_{scat}\left( \mu_{x}, LCP \right)=C_{0}\omega^{4}\left| \mu_{x}\left( LCP \right) \right|^{2}=C_{1}\left( \omega\right)\left| L_{y}\left( \omega\right)L_{em}\left( \omega\right)-4\omega^{2}g_{y}^{2}+4i\omega^{2}g_{x}g_{y} \right|^{2}$$

$$\begin{aligned} =C_{1}\left( \omega\right)\left( C_{2}\left( \omega\right)+8\omega^{2}g_{x}g_{y}Imag\left( L_{y}\left( \omega\right)L_{em}\left( \omega\right) \right) \right)\#\left( S9 \right) \end{aligned}$$

$$\begin{aligned} Q_{scat}\left( \mu_{x},RCP \right)=C_{1}\left( \omega\right)\left( C_{2}\left( \omega\right)-8\omega^{2}g_{x}g_{y}Imag\left( L_{y}\left( \omega\right)L_{em}\left( \omega\right) \right) \right)\#\left( S10 \right) \end{aligned}$$

$$\begin{aligned} Q_{scat}\left( \mu_{y},LCP \right)=C_{1}\left( \omega\right)\left( C_{3}\left( \omega\right)-8\omega^{2}g_{x}g_{y}Imag\left( L_{x}\left( \omega\right)L_{em}\left( \omega\right) \right) \right)\#\left( S11 \right) \end{aligned}$$

$$\begin{aligned} Q_{scat}\left( \mu_{y},RCP \right)=C_{1}\left( \omega\right)\left( C_{3}\left( \omega\right)+8\omega^{2}g_{x}g_{y}Imag\left( L_{x}\left( \omega\right)L_{em}\left( \omega\right) \right) \right)\#\left( S12 \right) \end{aligned}$$

where

$$C_{1}\left( \omega\right)=\frac{C_{0}\omega^{4}F_{0}^{2}}{\left| L_{x}\left( \omega\right)L_{y}\left( \omega\right)L_{em}\left( \omega\right)-4L_{x}{(\omega)\omega}^{2}g_{y}^{2}-4L_{y}\left( \omega\right)\omega^{2}g_{x}^{2} \right|^{2}}$$

$$C_{2}\left( \omega\right)=\left| L_{y}\left( \omega\right)L_{em}\left( \omega\right) \right|^{2}-8\omega^{2}g_{y}^{2}Real\left( L_{y}\left( \omega\right)L_{em}\left( \omega\right) \right)+16\omega^{4}g_{y}^{2}(g_{x}^{2}+g_{y}^{2})$$

$$C_{3}\left( \omega\right)=\left| L_{x}\left( \omega\right)L_{em}\left( \omega\right) \right|^{2}-8\omega^{2}g_{x}^{2}Real\left( L_{x}\left( \omega\right)L_{em}\left( \omega\right) \right)+16\omega^{4}g_{x}^{2}(g_{x}^{2}+g_{y}^{2})$$

Therefore,

$$CDS=\frac{\left( Q_{scat}\left( \mu_{x},LCP \right)+Q_{scat}\left( \mu_{y},LCP \right) \right)-\left( Q_{scat}\left( \mu_{x},RCP \right)+Q_{scat}\left( \mu_{y},RCP \right) \right)}{\left( Q_{scat}\left( \mu_{x},LCP \right)+Q_{scat}\left( \mu_{y},LCP \right) \right)+\left( Q_{scat}\left( \mu_{x},RCP \right)+Q_{scat}\left( \mu_{y},RCP \right) \right)}$$

$$\begin{aligned} =\frac{8\omega^{2}g_{x}g_{y}}{C_{2}\left( \omega\right)+C_{3}\left( \omega\right)}Imag\left( \left( L_{y}\left( \omega\right)-L_{x}\left( \omega\right) \right)L_{em}\left( \omega\right) \right) \\ =g_{x}g_{y}\left( \left( \omega_{x}^{2}-\omega_{y}^{2} \right)\Gamma_{em}+\left( \omega_{em}^{2}-\omega^{2} \right)\left( \Gamma_{x}-\Gamma_{y} \right) \right)/\tilde{A}\#\left( S13 \right) \end{aligned}$$

which is the main text Equation (6). It can be clearly seen that breaking the degeneracy of cavity *x* and *y* modes is essential for a far-field chiral response.

In Equation (S13), the demonimator is:

$$\tilde{A}=\frac{\left| L_{y}\left( \omega\right)L_{em}\left( \omega\right) \right|^{2}+\left| L_{x}\left( \omega\right)L_{em}\left( \omega\right) \right|^{2}}{8\omega^{2}}-\left( g_{y}^{2}Real\left( L_{y}\left( \omega\right)L_{em}\left( \omega\right) \right)+g_{x}^{2}Real\left( L_{x}\left( \omega\right)L_{em}\left( \omega\right) \right) \right)+2\omega^{2}\left( g_{x}^{2}+g_{y}^{2} \right)^{2}$$

It can be seen that $\tilde{A}$ has a component that proportional to $g^{4}$, which reduces the magnitude of CDS at strong coupling conditions. Physically, this comes from the fact that only a sub-component of the plasmon mode *x* or *y* that is independent of the coupling strength is interfering with the cross-coupling term from the other orthogonally-polarized plasmon mode through the emitter. As shown in Equation (S6) and (S7), the cross-coupling terms in the numerator $\pm4i\omega^{2}g_{x}g_{y}$ are purely imaginary, so it cannot interfere with the $-{4\omega}^{2}g_{y}^{2}$ term which is purely real, and only interferes with the imaginary part of the term $L_{y}\left( \omega\right)L_{em}\left( \omega\right)$ which does not depend on the coupling strength.

S14 Discrepancy in scattering spectra between numerical simulations and theoretical models

The simulated scattering spectra show noticeable differences in the magnitudes of the high- and low-energy modes (main text Figure 2 and Figure S5), while their magnitudes are similar in the theoretical results (main text Figure 3(c) and (f)). Here we show that this discrepancy is largely due to the dispersion of silver permittivity which is accounted for in numerical simulations but neglected in the chiral-COM/JCM models. In Figure S13, we show the scattering spectra of the NBoM-emitter hybrid structure with either realistic dispersive silver permittivity (top panels) or artificial non-dispersive silver permittivity fixed at its value at 655 nm (bottom panels). With realistic dispersive permittivity, the difference in magnitudes of the low- and high-energy modes grows with the oscillator strengths and becomes significant for *f*=0.15. However, with artificial non-dispersive permittivity, the two modes have similar magnitudes for both *f*=0 and *f*=0.15. Since the theoretical models do not account for the dispersion of silver permittivity, their results show similar mode magnitudes.


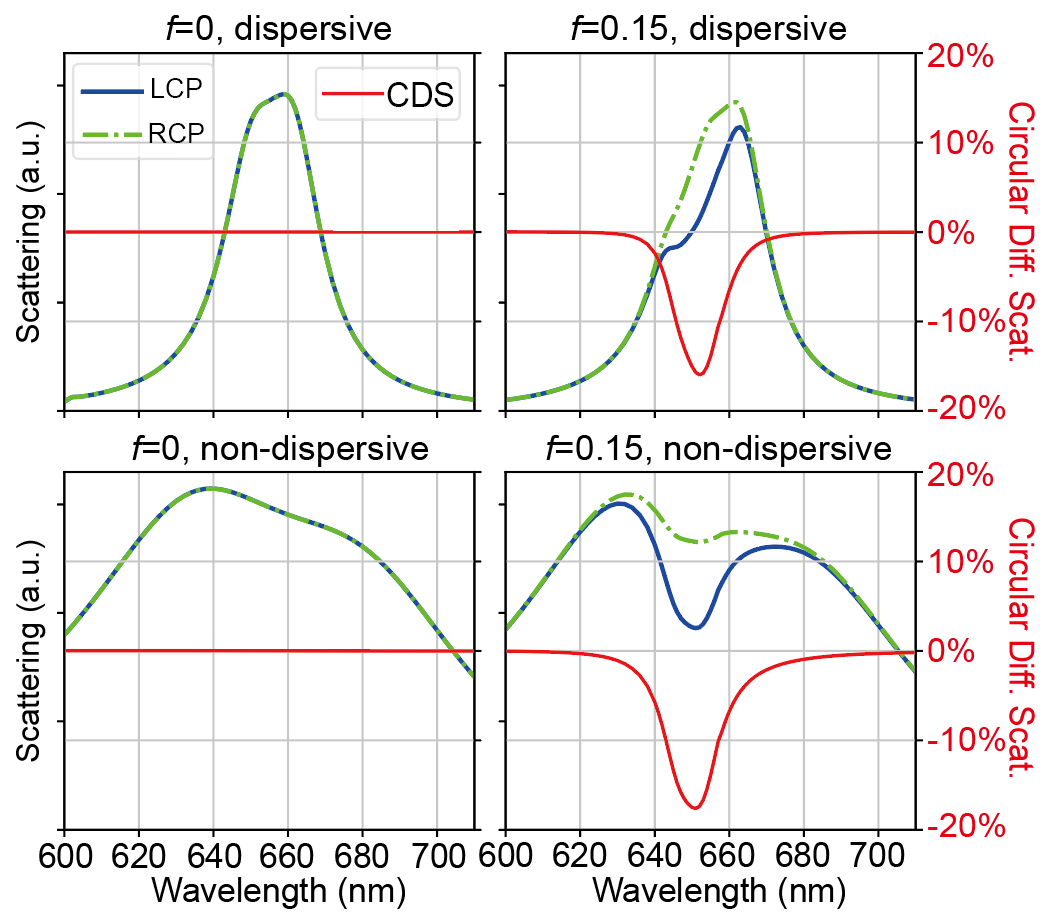


**Figure S13:** Effect of silver dispersion on the scattering and CDS spectra.

S15 Extinction spectra calculated by chiral-COM

According to Equations (4) and (5) in the main text:

$$\begin{aligned} \dot{\mu}_{x}(LCP, RCP)={-i\omega F}_{0}\frac{L_{y}\left( \omega\right)L_{em}\left( \omega\right)-{4\omega}^{2}g_{y}^{2}\pm4i\omega^{2}g_{x}g_{y}}{L_{x}\left( \omega\right)L_{y}\left( \omega\right)L_{em}\left( \omega\right)-{4L}_{x}\left( \omega\right)\omega^{2}g_{y}^{2}-4L_{y}\left( \omega\right)\omega^{2}g_{x}^{2}}e^{-i\omega t}\#\left( S14 \right) \end{aligned}$$

$$\begin{aligned} \dot{\mu}_{y}(LCP, RCP)={\pm\omega F}_{0}\frac{L_{x}\left( \omega\right)L_{em}\left( \omega\right)-{4\omega}^{2}g_{x}^{2}\mp4i\omega^{2}g_{x}g_{y}}{L_{x}\left( \omega\right)L_{y}\left( \omega\right)L_{em}\left( \omega\right)-{4L}_{x}\left( \omega\right)\omega^{2}g_{y}^{2}-4L_{y}\left( \omega\right)\omega^{2}g_{x}^{2}}e^{-i\omega t}\#\left( S15 \right) \end{aligned}$$

where the $\pm$ sign indicate LCP and RCP, respectively. The extinction is proportional to the time-averaged work done by the external force:

$$Q_{ext}\propto\left\langle Real\left( F_{x} \right)Real(\dot{\mu}_{x})+{Real(F}_{y})Real(\dot{\mu}_{y} \right\rangle$$

$$\begin{aligned} \left\langle Real\left( F_{x} \right)Real(\dot{\mu}_{x}) \right\rangle\\ =\left\langle\omega F_{0}^{2}\cos\left( \omega t \right)\cdot Imag\left( \frac{L_{y}\left( \omega\right)L_{em}\left( \omega\right)-{4\omega}^{2}g_{y}^{2}\pm4i\omega^{2}g_{x}g_{y}}{L_{x}\left( \omega\right)L_{y}\left( \omega\right)L_{em}\left( \omega\right)-{4L}_{x}\left( \omega\right)\omega^{2}g_{y}^{2}-4L_{y}\left( \omega\right)\omega^{2}g_{x}^{2}}e^{-i\omega t} \right) \right\rangle\#\left( S16 \right) \end{aligned}$$

$$\begin{aligned} \left\langle Real\left( F_{y} \right)Real(\dot{\mu}_{y}) \right\rangle\\ =\left\langle\omega F_{0}^{2}\sin\left( \omega t \right)\cdot Real\left( \frac{L_{x}\left( \omega\right)L_{em}\left( \omega\right)-{4\omega}^{2}g_{x}^{2}\mp4i\omega^{2}g_{x}g_{y}}{L_{x}\left( \omega\right)L_{y}\left( \omega\right)L_{em}\left( \omega\right)-{4L}_{x}\left( \omega\right)\omega^{2}g_{y}^{2}-4L_{y}\left( \omega\right)\omega^{2}g_{x}^{2}}e^{-i\omega t} \right) \right\rangle\\ =\left\langle\omega F_{0}^{2}\cos\left( \omega t \right)\cdot Imag\left( \frac{L_{x}\left( \omega\right)L_{em}\left( \omega\right)-{4\omega}^{2}g_{x}^{2}\mp4i\omega^{2}g_{x}g_{y}}{L_{x}\left( \omega\right)L_{y}\left( \omega\right)L_{em}\left( \omega\right)-{4L}_{x}\left( \omega\right)\omega^{2}g_{y}^{2}-4L_{y}\left( \omega\right)\omega^{2}g_{x}^{2}}e^{-i\omega t} \right) \right\rangle\#\left( S17 \right) \end{aligned}$$

Therefore,

$$Q_{ext}\propto\left\langle Real\left( F_{x} \right)Real(\dot{\mu}_{x})+{Real(F}_{y})Real(\dot{\mu}_{y}) \right\rangle$$

$$\begin{aligned} =\left\langle\omega F_{0}^{2}\cos\left( \omega t \right)\cdot Imag\left( \frac{{L_{y}\left( \omega\right)L_{em}\left( \omega\right)-{4\omega}^{2}g_{y}^{2}+L}_{x}\left( \omega\right)L_{em}\left( \omega\right)-{4\omega}^{2}g_{x}^{2}}{L_{x}\left( \omega\right)L_{y}\left( \omega\right)L_{em}\left( \omega\right)-{4L}_{x}\left( \omega\right)\omega^{2}g_{y}^{2}-4L_{y}\left( \omega\right)\omega^{2}g_{x}^{2}}e^{-i\omega t} \right) \right\rangle\#\left( S18 \right) \end{aligned}$$

As can be seen, the cross-coupling terms in $\mu_{x}$ and $\mu_{y}$ cancel each other in the overall extinction formula, so the extinction spectra are identical for LCP and RCP excitations. Figure S14 shows the extinction spectra for four different coupling strengths. While the mode splitting gets larger with increasing coupling strength, there is always no difference between the LCP and RCP extinction spectra.


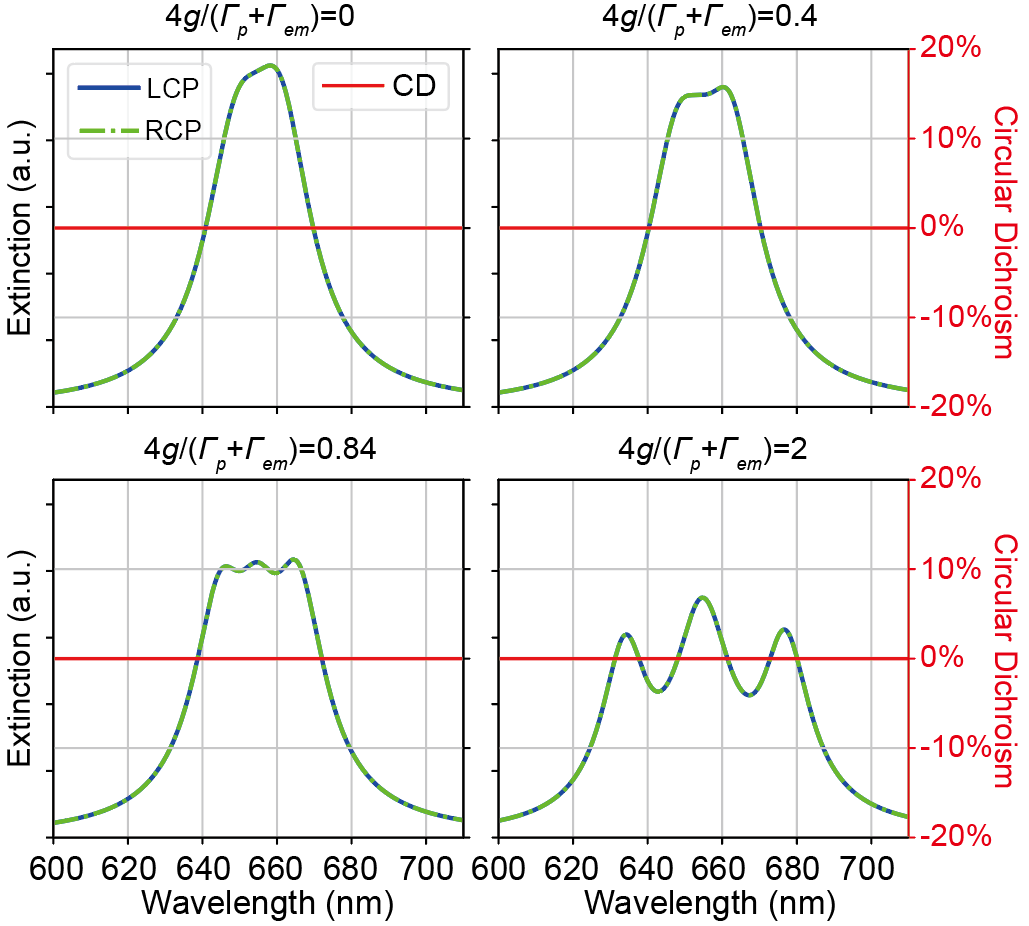


**Figure S14:** Extinction and its CD spectra under LCP/RCP excitations and the $\rho_{CP}$ spectra for different coupling strengths calculated by chiral-COM.

S16 Manipulating CDS by changing the gap property

Our theory for the CDS provides a general guideline for manipulating the CDS by tuning the plasmonic nanocavities as well as the plasmon-emitter coupling strength. The NBoM nanocavity, belonging to a family of metal-insulator-metal nanogap structures, are known to be highly sensitive to nanogap properties. The CDS of the NBoM-emitter hybrid structure should be tunable changing the optical properties of the dielectric spacer. In Figure S15, we changed the spacer refractive index from 1.60 to 1.40, resulting in a shift of the plasmon modes scanning across the emitter energy, and studied the system CDS in different coupling regimes. For weak coupling (Figure S15(a)-(c)), the dispersion of the two overlapping plasmon modes follows the behavior of uncoupled modes, and the magnitude of $\rho_{CP}$ is relatively small. Upon increasing the coupling strength to the intermediate regime (Figure S15(d)-(f)), an anti-crossing feature emerges in the scattering maps and $\rho_{CP}$ is significantly enhanced, exceeding -20%. When we tune the system into the strong coupling regime (Figure S15(g-i)), three branches and a clear anti-crossing feature can be observed in the scattering. Moreover, the $\rho_{CP}$ map shows two branches with anti-crossing behavior. This demonstrates that the CDS of the hybrid system can be manipulated by changing the nanocavity and coupling properties. The optimal CDS can be attained for intermediate coupling when the emitter energy level lies between the energy of the two plasmon modes.


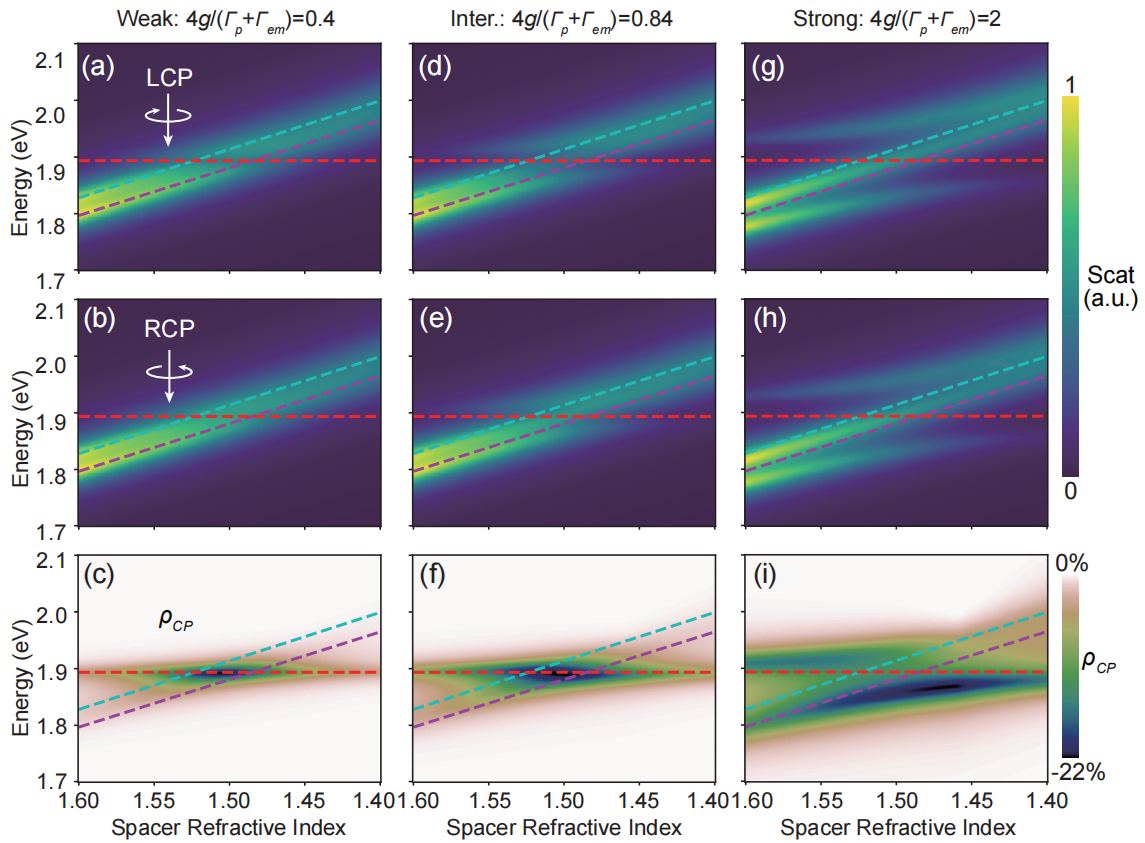


**Figure S15:** Tunability of the CDS by the spacer refractive index in different coupling regimes. Scattering and CDS spectra for different dielectric spacer refractive indexes for normalized coupling strengths $4g/(\Gamma_{p}+\Gamma_{em})$ of (a)-(c) 0.4 (weak coupling), (d)-(f) 0.84 (intermediate coupling), and (g-i) 2 (strong coupling).

S17 Angular distribution of chiral scattering DCP

The angular distribution of the chiral scattering DCP has a very similar behavior with that of the CDS, since they both originate from the extrinsic chirality. As shown in Figure S16(a), with a bare NBoM antenna (*f*=0), the overall integral scattering DCP spectrum remains zero. Nevertheless, due to the extrinsic chirality of the nanocavity, the scattering DCP can be nonzero and reach a magnitude close to 1 at oblique collection angles along the 45^o^, 135^o^, 225^o^ and 315^o^ azimuthal directions (Figure S16(b)). Again, for collection angles along the 0^o^ and 180^o^ azimuthal directions, the mirror symmetry still exists, leading to zero scattering DCP. When the emitter was present with the oscillator strength *f*=0.05, the scattering DCP shows a bisignate lineshape. At the short-wavelength side (point C_1_), the integral DCP is negative, and the angular distribution map shows a dominant negative region (Figure S16(c1)). At the long-wavelength side (point C_2_), the integral DCP is positive, and the angular distribution map shows a dominant positive region (Figure S16(c2)). As the emitter oscillator strength reaches *f*=0.2, there is a notable increase in the magnitude of the integral scattering DCP. Additionally, both the magnitude and size of the predominant DCP region in the angular distribution map show a corresponding growth (Figure S16(d1) and (d2)).


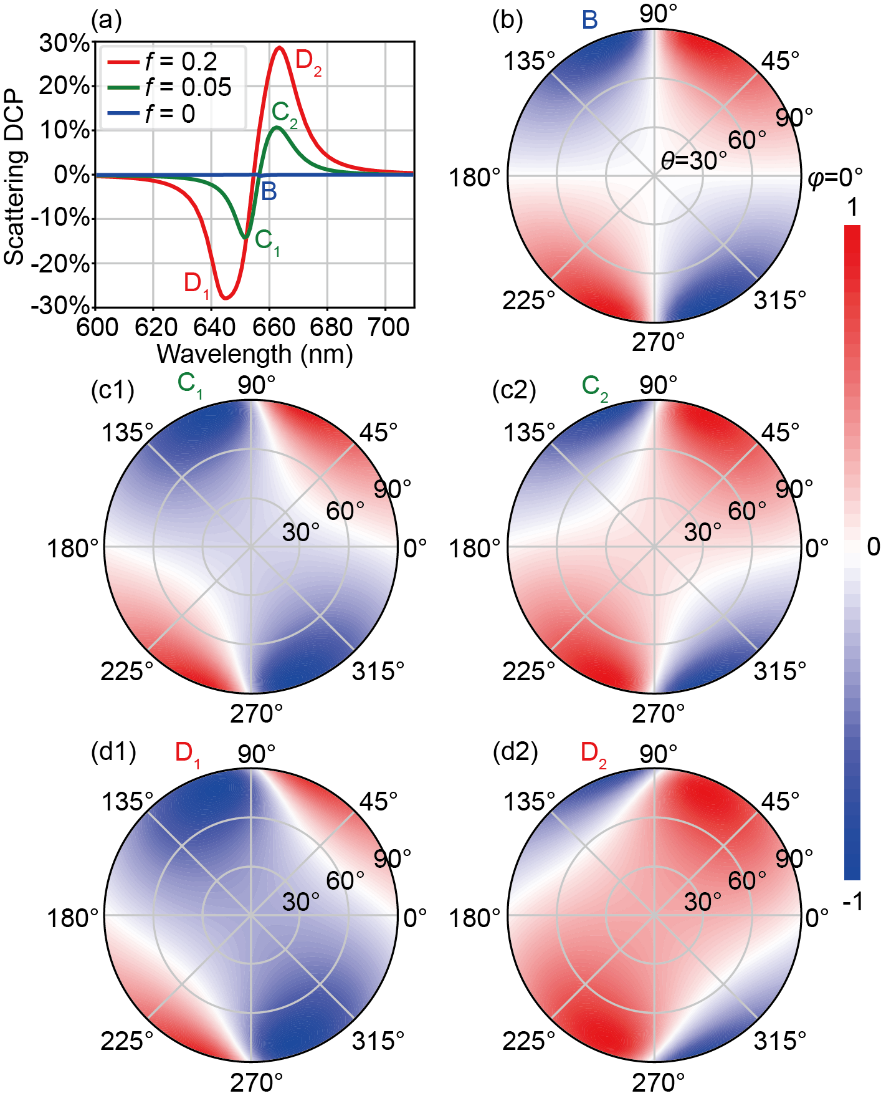


**Figure S16:** Angular distribution of chiral scattering DCP. (a) Overall integral scattering DCP for different emitter oscillator strengths. (b-d) Angular distribution of chiral scattering DCP for (b) *f*=0, (c1) and (c2) *f*=0.05, and (d1) and (d2) *f*=0.2. Here, in the polar plots, the radial axis corresponds to the polar angle $\theta$, the angular axis corresponds to the azimuthal angle $\varphi$, and the colormap represents the intensity distribution. The DCP mapping shows the DCP of scattered light along different directions under *x*-linearly-polarized excitation.

S18 Simulated radiative LDOS for LCP/RCP light

**
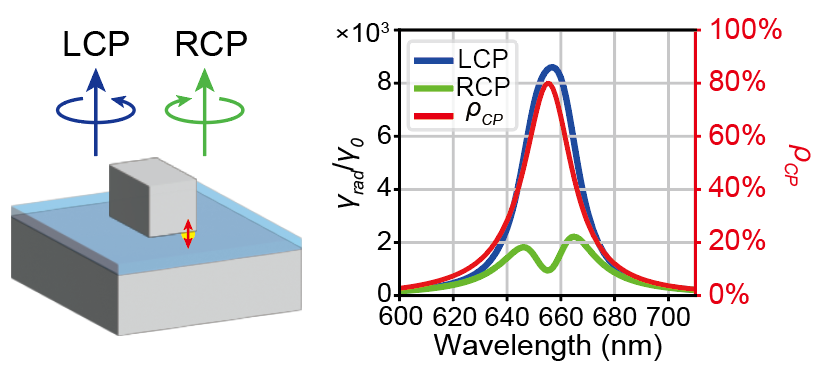
**

**Figure S17:** Simulated spectra of LCP/RCP radiative LDOS enhancement $\gamma_{rad}/\gamma_{0}$ and its DCP.

Figure S17 shows the simulated spectra of LCP/RCP radiative LDOS enhancement $\gamma_{rad}/\gamma_{0}$ and DCP $\rho_{CP}=(\gamma_{rad,L}-\gamma_{rad,R})/(\gamma_{rad,L}+\gamma_{rad,R})$ when an oscillating *z*-polarized electric point dipole is placed at the emitter position. The maximal enhancement for LCP/RCP radiative LDOS reaches over 8000/2000 respectively. The luminescence DCP is over 80%, in agreement with the circular differential ${|\boldsymbol{E}/\boldsymbol{E}_{\boldsymbol{0}}|}^{2}$ shown in the main text Figure 1(f) based on the principle of reciprocity.

S19 Angular distribution of chiral luminescence DCP

Figure S18 shows the angular distribution of the LCP/RCP components of the emitter luminescence. The LCP component of the luminescence is bright with an almost azimuthal-angle-independent emission pattern dominantly along the surface normal direction (*θ* = 0). Interestingly, the RCP component has a two-lobes emission pattern with dominant energy at polar angle $\theta\approx60^{\circ}$ and azimuthal angle $\varphi\approx30^{\circ}/210^{\circ}$ (this could be related to the position of the emitter). Moreover, the RCP component is much weaker than the LCP (RCP intensity was artificially magnified by 5 times for comparison). Therefore, the luminescence DCP is almost constant and remains at a high level across a wide range of emission directions, with only a small distortion due to the weak angle-dependent RCP luminescence. This can be advantageous for the realization of robust on-chip chiral photonic devices.


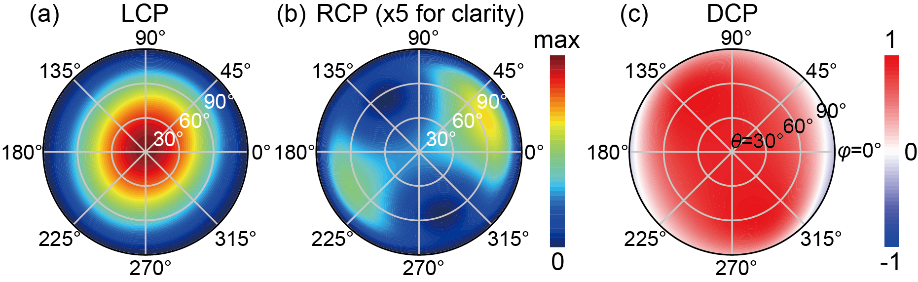


**Figure S18:** Angular distribution of the (a) LCP / (b) RCP components of the chiral luminescence and (c) the corresponding DCP. Here, in the polar plots, the radial axis corresponds to the polar angle $\theta$, the angular axis corresponds to the azimuthal angle $\varphi$, and the colormap represents the intensity distribution. Note that the intensity was magnified for 5 times in the RCP polar plot for clarity.

S20 Evolution of the oscillator dipole moments for chiral-COM

Figure S19 shows the time evolution of the three dipole moments calculated with chiral-COM, showing a similar trend with the chiral-JCM results (main text Figure 5(b)-(d)). Within the rotating-wave approximation, the JCM neglects the high-frequency oscillation and captures the envelope of the dipole magnitudes.


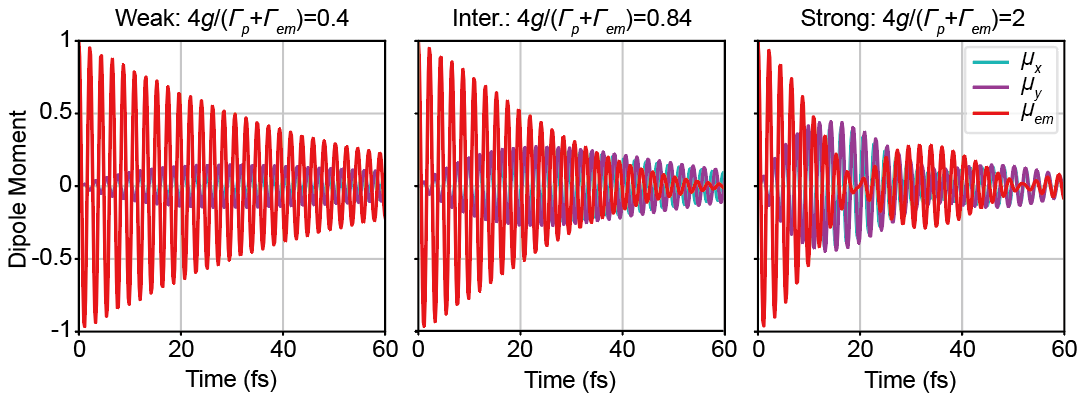


**Figure S19:** Evolution of dipole moments for different coupling strengths calculated by chiral-COM.

S21 Independency of the luminescence DCP on the coupling strength

The independency of the luminescence DCP on the coupling strength can be intuitively explained by the COM. For luminescence, the external force does not drive the plasmonic modes but the emitter:

$$\begin{aligned} \ddot{\mu}_{x}+\Gamma_{x}\dot{\mu}_{x}+\omega_{x}^{2}\mu_{x}=-{2g}_{x}\dot{\mu}_{em} \#\left( S19 \right) \end{aligned}$$

$$\begin{aligned} \ddot{\mu}_{y}+\Gamma_{y}\dot{\mu}_{y}+\omega_{y}^{2}\mu_{y}=-{2g}_{y}\dot{\mu}_{em} \#\left( S20 \right) \end{aligned}$$

$$\begin{aligned} \ddot{\mu}_{em}+\Gamma_{\mathrm{em}}\dot{\mu}_{em}+\omega_{em}^{2}\mu_{em}={2g}_{x}\dot{\mu}_{x}+{2g}_{y}\dot{\mu}_{y}+F_{em} \#\left( S21 \right) \end{aligned}$$

In experiments, the emitter is usually driven incoherently through photons (photoluminescence), electrons (cathodoluminescence), or electric currents (electroluminescence). The chiral luminescence originates from the relative phase difference ($\pm\pi/2$) between two oscillating dipoles $\mu_{x}$ and $\mu_{y}$ along the *x* and *y* directions, respectively. Namely, for pure circularly-polarized luminescence, the ratio between $\mu_{x}$ and $\mu_{y}$ should be $\pm i$. If we look at the oscillation component at any single frequency, we can easily obtain through Equations (S19) and (S20):

$$\begin{aligned} \beta=\frac{\mu_{y}\left( \omega\right)}{\mu_{x}\left( \omega\right)}=\frac{g_{y}\left( \omega_{x}^{2}-\omega^{2}-i\Gamma_{x}\omega\right)}{g_{x}\left( \omega_{y}^{2}-\omega^{2}-i\Gamma_{y}\omega\right)}\#\left( S22 \right) \end{aligned}$$

Here, $\beta$ is the complex ratio between the dipole moments $\mu_{y}$ and $\mu_{x}$. While the absolute luminescence intensity depends on the magnitudes of $\mu_{x,y}$, both the angular distribution and overall integral of luminescence DCP are solely decided by the complex ratio $\beta$:

$$\begin{aligned} DCP=\frac{\left| \mu_{x}-i\mu_{y} \right|^{2}-\left| \mu_{x}+i\mu_{y} \right|^{2}}{\left| \mu_{x}-i\mu_{y} \right|^{2}+\left| \mu_{x}+i\mu_{y} \right|^{2}}=\frac{2Imag\left( \beta\right)}{1+\left| \beta\right|^{2}}\#\left( S23 \right) \end{aligned}$$

For pure circularly-polarized luminescence ($DCP=\pm1$), the optimal $\beta$ values should be close to $\pm i$. Therefore, $\beta$ can serve as a figure of merit for the luminescence DCP. More specifically, since the luminescence DCP is solely dependent on the complex ratio $\beta$, it does not depend on the absolute magnitude of coupling strength $g$ but on the ratio $g_{x}/g_{y}$. In general system including ours, when the emitter oscillator strength or dipole orientation direction is changed, $g_{x}$ and $g_{y}$ will change proportionally, leaving the ratio $\beta$ unchanged. Therefore, neither the angular distribution nor integral luminescence DCP depends on the coupling strength.

S22 Application of the theoretical model to other plasmonic nanocavity-emitter systems

To further demonstrate the correctness and generality of our theoretical model, we apply it to two other plasmonic nanocavity-emitter coupled systems reported in previous literatures. As shown in Table S1, we successfully reproduced the experimentally measured chiral luminescence DCP with great accuracy.

| Ref. | Nanostructure | Figure | Reported DCP | $\beta$ | Our theory  (analytical) |
| --- | --- | --- | --- | --- | --- |
| [8] | Au rectangular plate | Fig. 3 | 93% | 0.334+0.841i | 93% |
| [9] | Al V-shape structure | Fig. 3d | 90% | 0.141+0.623i | 89% |

**Table S1:** Application of the theoretical model to other plasmonic nanocavity-emitter systems.

Here we briefly introduce the reproduction procedure. First, we calculated the quasinormal modes (QNM) of the plasmonic nanostructures based on the approach developed in [10], and obtained the mode energies, damping rates, as well as the vacuum electric field at the emitter position. The vacuum electric field is proportional to the coupling strength. For cathodoluminescence experiments [8,9], we assumed the electron beam to be an emitter located 5 nm above the nanostructure top surface. We have tested the results against different emitter locations and found that the final DCP is insensitive to the emitter vertical position. Next, we input the obtained parameters into the COM (Supplementary Material S2) and simulated the LCP and RCP components of the luminescence. Finally, the chiral luminescence DCP can be computed as $DCP=(I_{L}-I_{R})/(I_{L}+I_{R})$. Equivalently, the DCP can be directly obtained from the complex ratio $\beta$ in main text Equation (8) and (9). Note that for these two nanostructures with a large degeneracy lifting, $\beta$ is compensated for the differences in the radiative damping rates for $\mu_{x}$ and $\mu_{y}$. Figure S20 shows the reproduction results of the gold rectangular nanostructure. Figure S20(a) and (b) show the vacuum electric field $\boldsymbol{E}_{\boldsymbol{vac}}$ distribution of the *x* and *y* plasmon modes, with which we can calculate the coupling strength $g_{x}$ and $g_{y}$. Based on the COM, the reproduced $I_{L}$, $I_{R}$ (Figure S20(c)) and DCP (Figure S20(d)) as functions of emitter location are in excellent agreement with the measured results in Figure 3 of the reference [8].


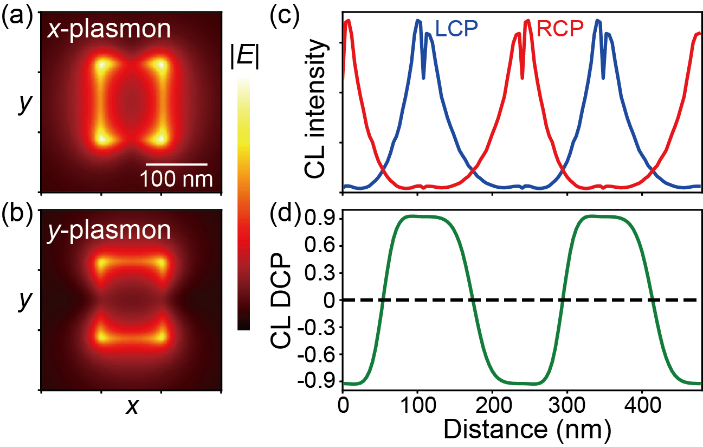


**Figure S20:** Reproduction of chiral luminescence of the rectangular nanostructure [8]. (a,b) Vacuum electric field distribution for the (a) *x* and (b) *y* plasmon mode at 5 nm above the nanostructure top surface. (c) LCP/RCP component and (d) DCP of the luminescence.

Likewise, through the complex ratio $\beta$, we can directly reproduce the DCP results of the aluminum V-shape nanostructure [9].


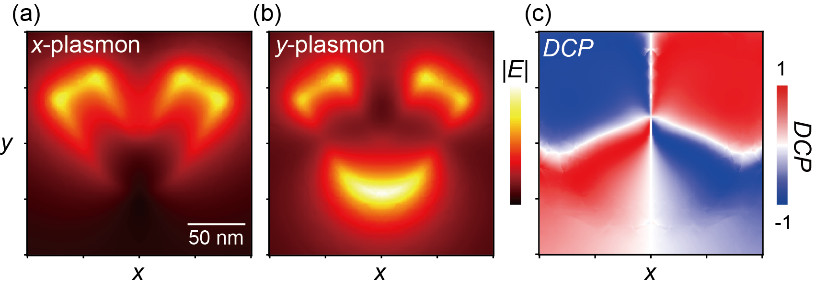


**Figure S21:** Reproduction of chiral luminescence of the V-shape nanostructure [9]. (a,b) Vacuum electric field distribution for the (a) *x* and (b) *y* plasmon mode at 5 nm above the nanostructure top surface. (c) DCP of the luminescence.

References

[1] J. Yang, J.-P. Hugonin, and P. Lalanne, "Near-to-Far Field Transformations for Radiative and Guided Waves," *ACS Photonics*, vol. 3, no. 3, pp. 395–402, 2016.

[2] P. B. Johnson and R. W. Christy, "Optical Constants of the Noble Metals," *Phys. Rev. B*, vol. 6, no. 12, pp. 4370–4379, 1972.

[3] X. Wu, S. K. Gray, and M. Pelton, "Quantum-dot-induced transparency in a nanoscale plasmonic resonator," *Opt. Express*, vol. 18, no. 23, pp. 23633, 2010.

[4] J. R. Johansson, P. D. Nation, and F. Nori, "QuTiP: An open-source Python framework for the dynamics of open quantum systems," *Computer Physics Communications*, vol. 183, no. 8, pp. 1760–1772, 2012.

[5] J. R. Johansson, P. D. Nation, and F. Nori, "QuTiP 2: A Python framework for the dynamics of open quantum systems," *Computer Physics Communications*, vol. 184, no. 4, pp. 1234–1240, 2013.

[6] H. Duan, A. I. Fernández-Domínguez, M. Bosman, S. A. Maier, and J. K. W. Yang, "Nanoplasmonics: Classical down to the Nanometer Scale," *Nano Lett.*, vol. 12, no. 3, pp. 1683–1689, 2012.

[7] V. R. Manfrinato, L. Zhang, D. Su, H. Duan, R. G. Hobbs, E. A. Stach, and K. K. Berggren, "Resolution Limits of Electron-Beam Lithography toward the Atomic Scale," *Nano Lett.*, vol. 13, no. 4, pp. 1555–1558, 2013.

[8] S. Zu, T. Han, M. Jiang, Z. Liu, Q. Jiang, F. Lin, X. Zhu, and Z. Fang, "Imaging of Plasmonic Chiral Radiative Local Density of States with Cathodoluminescence Nanoscopy," *Nano Lett.*, vol. 19, no. 2, pp. 775–780, 2019.

[9] S. Zu, T. Han, M. Jiang, F. Lin, X. Zhu, and Z. Fang, "Deep-Subwavelength Resolving and Manipulating of Hidden Chirality in Achiral Nanostructures," *ACS Nano*, vol. 12, no. 4, pp. 3908–3916, 2018.

[10] W. Yan, R. Faggiani, and P. Lalanne, "Rigorous modal analysis of plasmonic nanoresonators," *Phys. Rev. B*, vol. 97, no. 20, pp. 205422, 2018.
